# "Antelope": a hybrid-logic model checker for branching-time Boolean GRN analysis

**DOI:** 10.1186/1471-2105-12-490

**Published:** 2011-12-22

**Authors:** Gustavo Arellano, Julián Argil, Eugenio Azpeitia, Mariana Benítez, Miguel Carrillo, Pedro Góngora, David A Rosenblueth, Elena R Alvarez-Buylla

**Affiliations:** 1 Centro de Ciencias de la Complejidad, piso 6, ala norte, Torre de Ingeniería, Universidad Nacional Autónoma de México, Coyoacán, 04510 México D.F., México; 2 Laboratorio de Genética Molecular, Desarrollo y Evolución de Plantas, Instituto de Ecología, Universidad Nacional Autónoma de México, 3er Circuito Universitario Exterior, Junto al Jardín Botánico, Coyoacán, 04510 México D.F., México; 3 Department of Functional Genomics and Proteomics, Masaryk University, Kotlářská 2, CZ-61137 Brno, Czech Republic; 4 CEITEC-Central European Institute of Technology, Masaryk University, Žerotínovo nám. 9, CZ-60177 Brno, Czech Republic; 5 Instituto de Investigaciones en Matemáticas Aplicadas y en Sistemas, Universidad Nacional Autónoma de México, Apdo. Postal 20-726, 01000 México D.F., México

## Abstract

**Background:**

In Thomas' formalism for modeling gene regulatory networks (GRNs), *branching time*, where a state can have *more than one possible future*, plays a prominent role. By representing a certain degree of unpredictability, branching time can model several important phenomena, such as (a) asynchrony, (b) incompletely specified behavior, and (c) interaction with the environment. Introducing more than one possible future for a state, however, creates a difficulty for ordinary simulators, because *infinitely many *paths may appear, limiting ordinary simulators to statistical conclusions. *Model checkers *for branching time, by contrast, are able to prove properties in the presence of infinitely many paths.

**Results:**

We have developed *Antelope *("Analysis of Networks through TEmporal-LOgic sPEcifications", http://turing.iimas.unam.mx:8080/AntelopeWEB/), a model checker for analyzing and constructing Boolean GRNs. Currently, software systems for Boolean GRNs use branching time almost exclusively for asynchrony. *Antelope*, by contrast, also uses branching time for incompletely specified behavior and environment interaction. We show the usefulness of modeling these two phenomena in the development of a Boolean GRN of the *Arabidopsis thaliana *root stem cell niche.

There are two obstacles to a direct approach when applying model checking to Boolean GRN analysis. First, ordinary model checkers normally only verify whether or not a *given *set of model states has a given property. In comparison, a model checker for Boolean GRNs is preferable if it *reports *the set of states having a desired property. Second, for efficiency, the expressiveness of many model checkers is limited, resulting in the inability to express some interesting properties of Boolean GRNs.

*Antelope *tries to overcome these two drawbacks: Apart from reporting the set of all states having a given property, our model checker can express, at the expense of efficiency, some properties that ordinary model checkers (e.g., NuSMV) cannot. This additional expressiveness is achieved by employing a logic extending the standard Computation-Tree Logic (CTL) with hybrid-logic operators.

**Conclusions:**

We illustrate the advantages of *Antelope *when (a) modeling incomplete networks and environment interaction, (b) exhibiting the set of all states having a given property, and (c) representing Boolean GRN properties with hybrid CTL.

## Background

### Gene regulatory network models

A major challenge in current biology is relating spatio-temporal gene expression patterns to phenotypic traits of an organism. These patterns result partly from complex regulatory interactions sustained principally by genes and encoded proteins. The complexity of such interactions exceeds the human capacity for analysis. Thus, mathematical and computational models of gene regulatory networks (GRNs) are indispensable tools for tackling the problem of mapping the genotype into the phenotype. These models have been fruitfully applied in numerous biological systems (e.g., [1-4]).

Within the various kinds of GRN models [5], Boolean GRNs are especially valuable for their simplicity and for nonetheless having a rich behavior yielding meaningful biological information [6,7]. Examples where Boolean GRNs have been successfully used are: the segment polarity gene network of *Drosophila melanogaster *[4,8], the flower organ determination GRN of *Arabidopsis thaliana *[9], the mammalian cell cycle [10], and the yeast cell cycle [11,12].

In a Boolean GRN, each gene has only two possible activation values: active (1) or inactive (0); intermediate expression levels are neglected. A *network state *at time *t *is a vector containing the activation values of all the genes in the GRN at time *t*. In addition, time is viewed as proceeding in discrete steps. The value of every gene *X *at time *t *+ 1 is specified by a Boolean function of the values of its regulators $g_{1},g_{2},\ldots ,g_{n_{X}}$ at time *t*.

### Branching time

Boolean GRNs are closely related to the formalism developed by Thomas and his collaborators [13-15]. Thus, computer systems for Boolean GRNs are often influenced by Thomas' formalism, which employs GRN models with branching time, allowing states with *more than one immediate future *[[13], p. 33]. A network state with more than one immediate future represents the fact that the next state of the regulatory system modeled by such a GRN can be any one of several states. Hence, the next state of the modeled system is only partially determined. Let us then say that there is an *indetermination *in the network. This indetermination in the system's behavior reflects a certain degree of unpredictability that can be identified with several important phenomena.

#### Asynchrony

**Figure 1:**
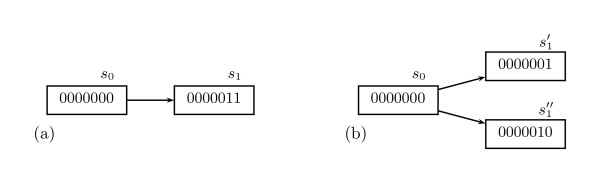
**A fragment of the state-transition graph of a Boolean GRN exemplifying asynchrony**. Assume that the behavior of a network specifies a simultaneous transition of the value of the two rightmost genes from 0 to 1 (panel (a)). If we exclude the possibility of simultaneous changes, it might be more realistic to model such a phenomenon with an indetermination (panel (b)).

One such phenomenon is *asynchrony *[[13], p. 33]. Experiments for inferring gene interaction do not normally establish the length of time between state changes. Hence, when such experiments indicate the change in value of two genes, say, it is preferable to model such a situation with a single state having two successors, one for each change, as illustrated in Figure 1. The reasons are that we do not know the relative values of both delays in real biological systems [[13], p. 44] and that complete synchrony might be practically impossible [[13], pp. 33, 55].

Many computer systems based on, or inspired by, Thomas' formalism (such as BooleanNet [16], BoolNet [17], GINsim [18-20], GNBox [21,22], SMBioNet [23,24], and SQUAD [25-27]) employ asynchronous models. Thomas' formalism, however, incorporates *two additional phenomena *with indeterminations, that are typically excluded in such systems.

#### Incompletely specified behavior

One such additional phenomenon is *incompletely specified behavior *[[13], p. 24]. This behavior may emerge, first, from a "synthetic" approach [[13], pp. 60-67], where we are interested in all Boolean GRNs having certain properties (e.g., a certain set of steady states) regardless of other properties. The tables specifying the network behavior would then have outputs whose value "does not matter" [[13], p. 24]. Second, lack of some of the experimental information of a regulatory system also emerges as incompletely specified behavior. In this case, the behavior tables would have outputs whose value we *do not know*.

#### Interaction with the environment

Another phenomenon usually neglected in computer systems for GRN analysis and that can be modeled with branching time is that of *interaction with the environment*. Assume that the next state of a regulatory system depends on the temperature: If the temperature is low, the system's next state will be one, but if the temperature is high, the system's next state will be different. Another example is the unpredictability of radiation-induced apoptosis [28]. In this case, for the same degree of radiation some cells will initiate apoptosis while others will not. Thomas and D'Ari reflect such an unpredictability with an "input variable" [[13], pp. 33-35] of an unknown value. This phenomenon can be readily incorporated with indeterminations.

### Simulators

Boolean GRNs are sometimes studied with *simulators *(e.g., Atalia [9], BooleanNet [16], and BoolNet [17]). A simulator attempts to replicate the behavior of a system by performing state changes in the *same order *as they occur in the system being modeled. Hence, network paths are traversed forward from one state to the next. In the presence of a state with more than one successor, such a straightforward approach must be complemented with additional mechanisms. Two of such mechanisms are: (a) a random device (randomly selecting one successor) and (b) backtracking (systematically selecting one successor after another by remembering which successors of each state have already been selected) coupled with a cycle-detection mechanism.

**Figure 2:**
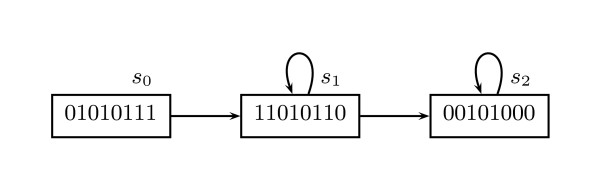
**A fragment of the state-transition graph of a Boolean GRN showing the appearance of infinitely many paths**. Infinitely many paths appear in this Boolean GRN because of one state (*s*_1_) having more than one future and occurring in a cycle. Some paths are: (*s*_0_*s*_1_*s*_2 _...), (*s*_0_*s*_1_*s*_1_*s*_2 _...), (*s*_0_*s*_1_*s*_1_*s*_1_*s*_2 _...), ... A simulator using a random device traverses the model forward, state by state, following a single path of the state graph, limiting the use of such a tool to drawing only statistical conclusions about all paths in models such as this one. Model checkers, by contrast, can prove precise properties, even in the presence of infinitely many paths resulting from states having more than one future.

A random device, on the one hand, allows for only drawing statistical conclusions. The reason is that in the presence of a state with more than one successor, the number of paths may be infinite [6], as depicted in Figure 2. Backtracking and cycle detection, on the other hand, can be inefficient (taking, in the worst case, an exponential amount of time in the size of the network [[29], p. 82]).

There are two important approaches for circumventing these difficulties. One of these techniques is an elaboration of backtracking so as to increase its efficiency by requiring certain *constraints *to be satisfied as the network is traversed [30]. The work by Corblin et al. [21,22] uses this approach. Another relevant method is model checking.

### Model checking

Model checking [31,32] is a collection of techniques for automatically verifying properties especially of discrete systems. The main ideas of model checking appeared 30 years ago [31,32]. At present, numerous model-checking tools exist. Model checking is routinely used, mainly for hardware verification, but also for software verification [33], and was distinguished with the A. M. Turing award in 2007. Model checking has been advocated for analyzing biological systems with increasing interest [6,24,34-43].

A model checker normally has as input (1) a "Kripke structure" representing a discrete system (comprising a finite number of states), (2) a distinguished "initial" state (or set of states) in the Kripke structure, and (3) a "temporal-logic" formula expressing a desirable property, that may or may not hold (i.e., be true) at a state. The output of the model checker is either a confirmation or a denial that the formula holds at the initial state(s) (given by the user as part of the input).

In a Kripke structure time is branching, so that there may be more than one possible future of a given state. The introduction of branching time may produce infinitely many forward traversals (see Figure 2). Model checkers, however, unlike simulators randomly selecting a successor state, can systematically analyze such infinitely many possibilities [6]. Intuitively, this is often done by traversing the Kripke structure in reverse and accumulating the set of all states at which a subformula holds. Model checking amounts, thus, to performing an exhaustive search (in the presence of branching time). Such a search plays the role of a *mathematical proof *establishing a property for infinitely many paths.

### Programming vs. formula writing

By being based on properties formalized in temporal logic, model checkers have another advantage over simulators. The decision of whether or not a state satisfies a property of interest is programmed in the simulator itself. Therefore, if an unforeseen property appears during the usage of a Boolean GRN simulator, such a property must be incorporated in the simulator by modifying program code. This renders simulators rigid: either the user's needs are anticipated or reprogramming must be done.

Compared with simulators, model checkers exhibit the benefit of having replaced programming with temporal-logic formula writing. Instead of having to modify the computer program of a simulator, many new queries can be dealt with by writing new temporal-logic formulas (as long as the queries can be expressed in the selected logic), which (unlike large programs and their modifications) are concise and self-contained.

### Organization of this paper

In the Implementation section, we first illustrate both Computation-Tree Logic (CTL) [31] and its hybrid extension, Hybrid CTL (which we based on [44,45]), chosen to be able to express interesting properties for Boolean GRN analysis and construction. The term "hybrid" here means a combination of propositional modal logic with classical predicate logic, and should not to be confused with hybrid model checking, combining discrete with continuous variables. The Implementation section subsequently covers the model-checking algorithms and some implementation details. Next we show, in the Results section, the use of the *Antelope *model checker in the presence of indeterminations either caused by environment interaction or by an incompletely specified behavior. Finally, the Discussion section reviews other similar software systems, compares *Antelope *with such systems, and outlines features planned for the future.

## Implementation

This section first covers the temporal logics used by *Antelope*. After explaining CTL, we turn our attention to its hybrid extension. Next, we cover the model-checking algorithms, as well as additional implementation issues.

### Computation-Tree Logic

We now give a short account of CTL and refer the reader to additional file 1 of this paper for a gentle introduction and to additional file 2 for a formal definition of CTL. More thorough treatments can be found in [46-50].

#### Boolean and temporal operators

Formulas in CTL can have Boolean operators, such as **not **and **or**. In addition, such formulas can have "temporal operators", allowing us to refer to formulas holding in the future of a particular state. In this case, we must indicate whether we mean some future or all futures. Hence, it is possible to refer either (1) to *some *path starting in the present with the "modality" **E**, or (2) to *all *paths starting in the present with the modality **A**. Similarly, it is possible to refer (a) to the immediate future with the modality **X**, (b) to any state in the present or any point in the future with the modality **F**, or (c) to all states in the present and in the future with the modality **G**. Table 1 summarizes these modalities.

**Table 1:** CTL modalities

*modality*	*meaning*
**E**	some path (i.e., there Exists a path)

**A**	All paths

**X**	neXt state (i.e., immediate future)
**F**	any state either in the present or in the Future
**G**	all states in the present and in the future (Global)

A *temporal operator *is composed of a modality in the upper part together with a modality in the lower part of this table, which results in six temporal operators. (Often more temporal operators are included in CTL [49].) For example, a formula asserting that there exists a path such that in the present or in the future *g*_0 _does not hold (i.e., *g*_0 _is inactive) and *g*_1 _does hold (i.e., *g*_1 _is active) would be: "**EF**((**not ***g*_0_) **and ***g*_1_)". Hence, assuming that there is a single state *s *in which *g*_0 _does not hold and *g*_1 _does hold, this formula can be used to obtain the basin of attraction of such a state, with a model checker computing all states at which a given formula holds. The formula "**AX **((**not ***g*_0_) **and ***g*_1_)" holds at all states from which it is *necessary *to reach *s *in one step, i.e., states which have *s *as their only next state. The formula "**EX **((**not ***g*_0_) **and ***g*_1_)" holds at all states from which it is *possible *to reach *s *in one step, i.e., states which have *s *as a next state (and possibly other next states because of indeterminations). Other CTL formulas can characterize, for instance, whether or not it is necessary to go through a state *s*_1 _to reach another state *s*_2_. See [51] for a list of CTL formulas specifying various biological properties.

#### Some properties not expressible in CTL

There do not exist, however, CTL formulas for characterizing steady states (i.e., a formula holding exactly at the set of all steady states of an arbitrary Boolean GRN) [51], or oscillations. This motivates the use of a more expressive logic than CTL. *Antelope *provides a "hybrid" extension of CTL.

### Hybrid Computation-Tree Logic

This subsection is devoted to Hybrid CTL. We refer the reader to additional file 1 of this paper for a gentle introduction and to additional file 2 for a formal definition of Hybrid CTL. Deeper treatments of hybrid logics are in [52,53].

#### State variables

The main idea behind the hybrid extension of a temporal logic consists in the addition of variables allowing us to refer to states (i.e., state variables). The downarrow binder "↓*σ*" sets the state variable *σ *to the current state of evaluation. The formula "↓*σ*.**AX ***σ*", for example, characterizes the set of states which have themselves as their only next state. Hence, Hybrid CTL allows us to characterize the set of steady states. Moreover, by employing branching time, we are able to distinguish between two kinds of steady state. When a state has only one transition from and to itself, following Thomas and D'Ari [13], we will call it a *stable steady state*. When a state has, in addition to a self-loop, other transitions going to other states, following [13], we will call it an *unstable steady state *(named "stationary" state in [51]). Hybrid CTL formulas for calculating both these sets of states are: "↓*σ*.**AX ***σ*", for the set of stable steady states, and "↓*σ*.**EX***σ*", for the union of the sets of stable and unstable steady states.

### Other formulas

#### Attractors of various sizes and oscillations

The notion of a steady state can be generalized in an *attractor*, possibly involving more than one state. A steady state would then be a one-state attractor. A formula characterizing attractors of any size would be: "↓*σ*.**EX EF ***σ*".

Another interesting formula would be "↓*σ*.**EX**((**not ***σ*) **and EX ***σ*)", which holds at states belonging to a size-two attractor. Oscillations, where a gene is alternatively active and inactive, can also be characterized in Hybrid CTL: Additional file 1 explains a formula for the basin of attraction of possible oscillations. We refer the reader to the *Antelope *web site http://turing.iimas.unam.mx:8080/AntelopeWEB/ for more formulas.

### Algorithms

#### CTL

*Antelope *uses a standard "labeling" algorithm [46] for ordinary CTL formulas. Labeling algorithms for model checking are so called because we can think of each state as being labeled with the subformulas holding at that state.

Say that the formula given by the user is *φ*. The labeling algorithm starts by considering the simplest subformulas of *φ*, that is, the names of the genes. For each gene *g*, labeling all states at which the formula "*g*" holds is easy, as that information is already present in the Kripke structure.

Next, the labeling algorithm proceeds to more complex subformulas, until *φ *is reached, by treating the operator of each such subformula by cases. For instance, if the subformula is of the form "*ψ*_1 _**and ***ψ*_2_", then the labeling algorithm computes the set of states at which such a subformula holds as the intersection of the set of states at which *ψ*_1 _holds with the set of states at which *ψ*_2 _holds. All Boolean operators can be treated by combining set operations, like union, intersection, and set difference.

The labeling algorithm treats some temporal operators, such as **AX**, by using equivalences. For example, "**AX ***ψ*" is equivalent to "**not EX not ***ψ*". The rest of the temporal operators, however, must be dealt with explicitly. For all such primitive operators the labeling algorithm traverses the Kripke structure in *reverse*. Take for instance "**EX ***ψ*", which holds if there Exists a neXt state at which *ψ *holds. Given the set of states at which *ψ *holds, the labeling algorithm treats "**EX ***ψ*" by obtaining all states which have an immediate successor in such a set, i.e., all the *predecessors *of the states in such a set. The labeling algorithm processes operators such as **EG **by repetitively traversing the Kripke structure in reverse.

#### Hybrid CTL

The labeling algorithm is efficient (taking polynomial time in the size of the Kripke structure). The additional expressiveness of hybrid operators, such as "↓" comes at a price, however. Given a CTL formula *φ*, the computation of the set of states at which a formula of the form "↓*σ*.*φ*" holds involves calling the labeling algorithm with *φ once for each state*. The decrease in efficiency is even more if the "↓" operator appears nested. *Antelope*, however, treats certain patterns in special ways, requiring less time than a direct approach.

### More implementation issues

*Antelope *is a symbolic model checker [54], representing state sets by Reduced, Ordered Binary-Decision Diagrams (BDDs) [55]. (In particular, *Antelope *employs JavaBDD [56], which in turn uses BuDDy [57].)

#### Representation of a set of states

A BDD is a representation of a Boolean function. Thus, to use a BDD for representing a set of states in a Kripke structure we must view such a set as a Boolean function. This is possible if each row of the truth table of the Boolean function corresponds to an element which may or may not belong to such a set. The value of such a function will be 1 at exactly those states belonging to the set.

#### Representation of a set of transitions

In addition to representing sets of states, BDDs are used for representing the set of transitions of Kripke structures. In this case, the Boolean function has twice as many variables as there are genes. The reason is that each transition (corresponding to a row in the truth table of such a function) has both a source and a terminating state. BDDs are often surprisingly concise, allowing the verification of many large Kripke structures, with more than 10^20 ^states [54]. We refer the reader to [49] for a detailed description of BDDs and their use in symbolic model checking.

#### Optimizations

Apart from the use of BDDs, *Antelope *has several "optimizations" (i.e., special treatment of particular patterns so as to increase the efficiency). For example, a straightforward formula characterizing the states with more than one successor has the pattern "↓*σ*.**EX **↓*τ*.*φ*". If evaluated as described in the Algorithms section, this formula would call the labeling algorithm a number of times proportional to the square of the number of states (*O*(|*S*|^2^), where |*S*| is the number of states). To find the set of states with more than one successor, however, it is not necessary to visit all states for each state of the Kripke structure. It suffices to be able to enumerate the successors of each state. *Antelope *treats the formula for characterizing the states with more than one successor as a special case so that the CTL model-checking algorithm is called with *φ *as input a number of times linear in the size of the Kripke structure (*O*(|*S*| + |*R*|), where |*R*| is the number of transitions).

Another optimization is that of the operator **EY **(for "Exists Yesterday"), which is the converse of **EX**. Although this operator need not be primitive, *Antelope *does treat it as primitive by simply traversing the transitions forward. This operator allows the user to view *Antelope *as a kind of simulator.

Additional file 3 has a table comparing the verification times for a few models with respect to some properties of increasing complexity.

#### Input formats

*Antelope *accepts two formats for describing the Boolean GRN: tables and equations. In both cases, the values of a gene (at the current time step) are specified as a Boolean *relation *which depends on the values of (some) genes (at the previous time step). A table can be viewed as an extension of an ordinary truth table, where stars are allowed on the right-hand side, denoting indeterminations. Sometimes, however, it may be more convenient to use a logical formula instead of a truth table. Hence, *Antelope *accepts equations, each of which is of the form:

$$X:=f_{X}\left(g_{1},g_{2},\ldots ,g_{n_{X}}\right)$$

where the left-hand side represents the value of the gene *X *at the current time step, and the right-hand side is an arbitrary Boolean function (defined employing the usual Boolean operators, such as conjunction, disjunction, or negation) on the values of genes at the previous time step. To be able to represent indeterminations, we need *two *equations with the same left-hand side. We refer the reader to the *Antelope *user's manual, which appears in additional file 4, and in the URL http://turing.iimas.unam.mx:8080/AntelopeWEB/.

## Results

We now exemplify the use of Antelope for analyzing Boolean variants of the *A. thaliana *root stem cell niche GRN. Stem cells or initials are undifferentiated cells from which particular cell types of the organisms are generated; the microenvironment in which stem cells are located is called the stem cell niche.

Anatomically, stem cell niches are conformed by two different cell types, the stem cells themselves, and another cell or group of cells sometimes generically called organizer cells [58]. The organizer cells maintain the stem cells in the undifferentiated state through short-range signals. Understanding how the different cells conforming stem cell niches are specified, as well as how the balance between cell division and cell differentiation is maintained in the niches, is central for understanding the development, growth and regeneration processes occurring in plants and animals. In particular, plant stem cell niches constitute valuable model systems for studying regenerative and plastic developmental processes, as these organisms grow new organs and structures throughout their life [58,59].

We focus on the root stem cell niche of *A. thaliana*, that is located near the root tip and is well characterized at the anatomical and molecular level (see the recent review in [60]). This niche is conformed by the so-called quiescent center (QC), which is in turn conformed by the organizer cells of the root SCN, and is surrounded by four different stem cell types [59]. Each of these four types of stem cell will give rise to a different cell lineage: vascular, cortex/endodermal, epidermal, and columella/root-cap cells. However, in this contribution two of the stem cell types (epidermal and root-cap cells) are considered as only one since the available experimental evidence is not enough to distinguish between them at the gene expression level (see more details in [61]), leaving only four types of initial cells (QC, vascular, cortex/endodermal (CEI), and epidermal/root-cap (CEpI) initials).

**Figure 3:**
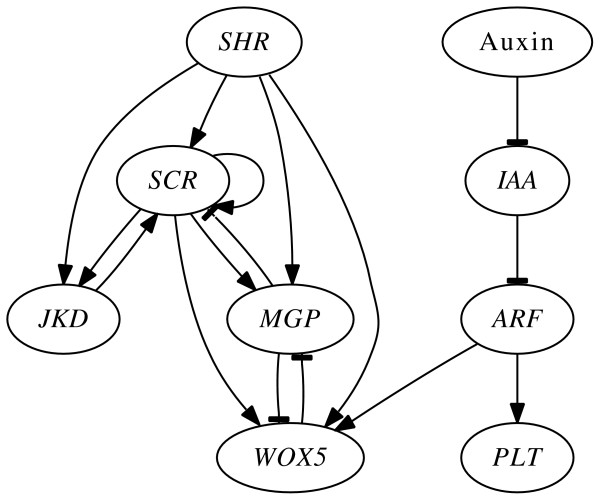
**The interaction diagram of the GRN underlying cell type determination in the root stem cell niche of the model plant A. thaliana**. The abbreviated names of the genes are inside ellipses and the edges correspond to the regulatory interactions. Auxin is a morphogene. The genes are: *Auxin*/*INDOLE-3-ACETIC ACID *(*Aux/IAA*), *AUXIN RESPONSE FACTOR *(*ARF*), *JACKDAW *(*JKD*), *MAGPIE *(*MGP*), *PLETHORA *(*PLT*), *SCARECROW *(*SCR*), *SHORTROOT *(*SHR*), and *WUSCHEL-RELATED HOMEBOX5 *(*WOX5*). Ordinary arrow heads denote activation; T-bar arrow heads denote inhibition.

Besides being thoroughly characterized at the anatomical level, the root stem cell niche of *A. thaliana *has been relatively well described from a molecular and genetic perspective. Indeed, some of the molecular components that are necessary to establish and maintain the root SCN cellular patterning have been recently uncovered. Among these components are the genes *SHORT-ROOT *(*SHR*) and its target gene *SCARECROW *(*SCR*), the immediately downstream genes of the dimer SHR/SCR, and other genes that interact with them. Another set of relevant genes includes the *PLETHORA *(*PLT*) genes, which have been proposed to be key components of the molecular readout of the plant hormone auxin. Finally, the QC specific gene *WUSCHEL RELATED HOMEOBOX5 *(*WOX5*) is fundamental for root SCN organization [60,62-64]; see the graphical representation of the interactions between these genes in Figure 3. Moreover, the expression patterns of these genes and the localization of their corresponding proteins have been described. Thus, it is possible to postulate a gene expression profile that characterizes each of the SCN cell types mentioned above according to the Table 2.

**Table 2:** Expected expression profiles for the cells conforming the A. thaliana root stem cell niche

Cell type	PLT	Auxin	ARF	Aux/IAA	SHR	SCR	JKD	MGP	WOX5
QC	1	1	1	0	1	1	1	0	1
Vascular	1	1	1	0	1	0	0	0	0
CEI	1	1	1	0	1	1	1	1	0
CepI	1	1	1	0	0	0	0	0	0

In order to define the rules for a Boolean GRN model for this system, we considered all the genes that have been reported to play a relevant role in the specification of the root stem cells and gathered the available experimental information for the regulation of their expression [60,61]; see Figure 3. These data included mostly molecular genetics experiments, such as experiments with plants containing a mutant allele of a gene. The resulting rules can be summarized in the following logical statements (uploaded in *Antelope *under the name 'Root gene regulatory network''):

// SHR; without regulators

// Auxin; without regulators

PLT: = ARF;

AUXINS: = AUXINS;

IAA: = ^~ ^AUXINS;

ARF: = ^~ ^IAA;

SHR: = SHR;

SCR: = SHR & SCR & (JKD | ^~^MGP);

JKD: = SHR & SCR;

MGP: = SHR & SCR &^~ ^WOX;

WOX: = ARF & SHR & SCR & (^~ ^MGP | WOX);

As has been proposed for other systems (e.g., [1,9]), we expected the stable steady states of our GRN model to correspond to the gene expression profiles characterizing the different stem cells within the root niche of *A. thaliana *(table above). Thus, from our knowledge of the system, we expected four stable steady states. The expected steady states are indeed obtained after postulating a mutual negative interaction between *WOX5 *and *MGP*, which gives rise to a new testable prediction [61].

Using this GRN model, we first illustrate the use of indeterminations representing incomplete experimental data. Next, we use indeterminations for modeling the influence of unpredictable external signals.

### Experimental gap

#### Steady states and *SCARECROW*

While developing the truth tables for this GRN, we detected an experimental gap. We know that *SCARECROW *(*SCR*), a target gene of the dimer SHORTROOT (SHR)/SCR [62,63], either loses or diminishes its own expression in the *JACKDAW *single mutant (*jkd*) in the stem cell niche [64]. The same is true for *SCR*-dependent quiescent-center marker *QC25 *[65]. The *MAGPIE *mutant (*mgp*), by contrast, has no visible phenotype. Finally, the *mgp jkd *double mutant recovers the *SCR *expression [64] (but see [66] for different results).

Based on this information, we established the truth table for *SCR*, which appears in Table 3. Observe the indetermination, reflecting the fact that activity could or could not be lost in a *jkd *background. *Antelope *produced three stable steady states, but four unstable steady states (see the Hybrid Computation-Tree Logic subsection for definitions of stable and unstable steady states). Hence, removing the indetermination in the above table may recover the four expected stable steady states. We performed the *jkd *loss-of-function simulation in our models to distinguish which of the two possibilities (i.e., no *SCR *transcription in *jkd *or *SCR *transcription in *jkd*) recovered the expected states. Interestingly, following the GRN state transitions *backwards*, using the **EX **operator, we noted that if *SCR *is unable to be expressed in *jkd*, then neither the *WUSCHEL-RELATED HOMEBOX5 *(*WOX5*) (another quiescent-center marker, dependent on *SCR *[60]) expression nor the *SCR *expression disappeared at the quiescent-center.

**Table 3:** Truth table for SCR

***SHR***	***SCR***	***JKD***	***MGP***	***SCR'***
	
0	0	0	0	0
0	0	0	1	0
0	0	1	0	0
0	0	1	1	0
0	1	0	0	0
0	1	0	1	0
0	1	1	0	0
0	1	1	1	0
1	0	0	0	0
1	0	0	1	0
1	0	1	0	0
1	0	1	1	0
1	1	0	0	1
1	1	0	1	*
1	1	1	0	1
1	1	1	1	1

Furthermore, our *jkd *mutant does cause a loss of the cortex-endodermis initials attractor, contrary to what is observed in experimental *jkd *mutants [64], suggesting that *jkd *only diminishes *SCR *expression. Again, following the GRN transitions backwards for the case in which *jkd *loss-of-function does not lose *SCR *expression, we found that the system was able to recover the *jkd *loss-of-function mutant. Based on the result found with the system including indeterminations, we replaced the star by a 1 in the table for *SCR*. Once the indetermination was so removed, we obtained four stable steady states.

### External signals

#### *FAS *and *SCR*

Let us now exemplify *Antelope *as used for modeling the effect of external signals that affect one or more GRN nodes. The root stem cell niche of *A. thaliana *is affected by several external signals, such as genes and molecules from modules involved in other processes in the organism. For example, Kaya and collaborators [67] reported that *FASCIATA1 *(*FAS1*) and *FASCIATA2 *(*FAS2*), hereafter collectively called *FAS*, affect *SCR *expression. In the *fas *mutant, *SCR *expression is deregulated and can be either expressed or not expressed in almost any cell of the root stem cell niche. Similarly, Inagaki and collaborators [68] reported the *TECHBI *(*TEB*) mutants also affecting *SCR *expression. Again, when *TEB *is mutated, *SCR *may or may not be expressed through the endodermal layer, the cortex-endodermis initial cells, and the quiescent center.

**Figure 4:**
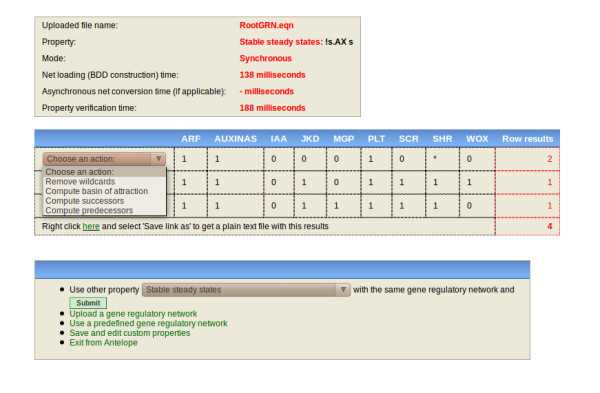
**Screenshot of Antelope showing the stable steady states for the stem cell niche GRN without indeterminations**. The upper frame displays the name of the file being analyzed, the analysis performed (with the Hybrid CTL formula), and the mode by which the property was checked (synchronous or asynchronous). The middle frame displays the analysis results. The bottom frame displays new actions that can be done. The stable steady states correspond to the root SCN cellular types.

**Figure 5:**
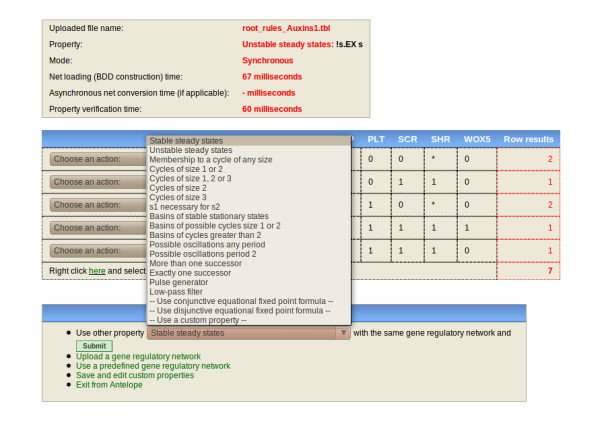
**Screenshot of Antelope showing the results for unstable steady state search for the stem cell niche GRN with an indetermination in the SCR logical rule**. This indetermination represents a mutation in *FAS*. As observed, *SCR *activity is present even in the absence of *SHR*, which is indispensable for *SCR *activity. It is important to note that some of the changes can only be observed analyzing the basins of attraction. For instance, the steady states for QC and CEI (two different cell types) are not possible without *SCR *presence; hence, a change of one steady state for another is only observable through their basins of attraction.

We incorporated *FAS *by adding a variable *FAS *to the truth table for *SCR*. For *FAS *= 1, the truth table obtained in the "Experimental gap" subsection was used. For *FAS *= 0, by contrast, *all the right-hand sides *of the new truth table had indeterminations. In the case of *TEB*, we only used indeterminations for the right-hand side of the *SCR *table where the output was 1 for the *teb *mutant. We found that under these conditions the original four attractors were preserved in both cases. We also found that in the *fas *mutant, *SCR *could be expressed in any of the four original attractors, while in the *teb *mutant *SCR *could or could not be expressed either in the quiescent center or in the cortex-endodermis attractor. It is worth noting that in both cases the basins of attraction changed. For instance, consider the states that without any indetermination originally led to the cortex-endodermis attractor. Such states could now lead to vascular initials due to *SCR *indeterminations, as expected given the experimental evidence. It is also important to note that even though *SCR *expression is clearly affected in real roots, cells may not switch among cell types. However, the results derived from modeling the GRN using *Antelope *are consistent with data currently available and demonstrate the utility of this tool when we deal with networks in which the truth tables for some genes are not completely known. Figures 4 and 5 show screenshots of this analysis.

### Other properties

These two analyses were based on indeterminations, stable and unstable steady states, and basins of attraction of such states. When designing and analyzing larger GRNs, more complex state attributes, such as global properties or conditional reachability may be useful.

For example, all the states occurring in either one-state or two-state attractors (which may be either stable or unstable) satisfy the formula "↓*σ*.**EX EX ***σ*". The formula "**EF**(↓*σ*.**EX EX ***σ*)", in turn, can be used to calculate the basins of attraction of all such attractors. Hence, the formula "**not**(**EF **(↓*σ*.**EX EX ***σ*))" would characterize the complement of all such basins of attraction. This is equivalent to the set of all states in the basins of attraction of attractors with more than two states. Similarly, the set of states occurring in exactly two-state attractors can be calculated with the formula "↓*σ*.**EX **((**not ***σ*) **and EX ***σ*)". These global properties cannot be expressed by CTL formulas.

Conditional reachability can be expressed with the **EU **operator (for "Exists Until"), a generalization of **EF**. Whereas "**EF***s*_2_" holds at all states from which it is possible to reach state *s*_2_, "**E**[*φ ***U ***s*_2_]" holds at all states from which it is possible to reach *s*_2 _by going only through states at which the formula *φ *holds. For instance, the set of states from which it is possible to reach *s*_2 _without going through *s*_1 _corresponds to the formula "**E**[(**not ***s*_1_) **U ***s*_2_]". By contrast, the set of states from which it is possible to reach *s*_2 _only by going through *s*_1 _at least once is the complement of the previous set of states with respect to the basin of attraction of *s*_2_: "**not**(**E**[(**not ***s*_1_) **U ***s*_2_]) **and EF ***s*_2_". In formulas having such schemata, we would need to name states. Such a naming is possible in CTL by identifying a state with the conjunction of its nonnegated active genes and its negated inactive genes. *Antelope*, by contrast, provides more concise ways of referring to a state, with a number which, if written in binary, follows the lexicographic order of the names of the genes. We refer the reader to the *Antelope *user's manual and site.

## Discussion

### Other related systems

We now describe other systems relevant for us. For brevity, we have to exclude certain works: First, we leave out Boolean GRN simulators, such as Atalia [9], BooleanNet [16], and BoolNet [17]. Second, we omit research based on structures other than Kripke structures; examples are: a work utilizing the LTL (Linear-time Temporal Logic) model checker of the Maude system [34], works using reactive modules with the Mocha model checker [42,43], and those employing probabilistic model checking with PRISM [35,37,38]. We start with systems based on Thomas' formalism and proceed with systems using continuous approaches.

#### GNBox

GNBox [21,22] applies constraint logic programming techniques [30] to Thomas' formalism [13]. Such a formalism establishes a search space resulting from states possibly having more than one successor. A straightforward implementation of a logic programming language (without constraints) typically traverses a search space following a depth-first, top-down discipline in the same way as an ordinary simulator. Unlike a simulator employing a random device, however, such an implementation utilizes *backtracking*. Observe that a depth-first, top-down discipline together with backtracking can take an exponential amount of time in the size of the model [[29], p. 82]. Constraint logic programming languages, nevertheless, use constraints to efficiently traverse the search space. In particular, GNBox expresses constraints as a Boolean satisfiability (SAT) problem that is turned over to a dedicated SAT solver. This approach is able to model many possible GRNs, thereby pruning the search space and eliminating the need for performing numerous simulations. By expressing desired properties as constraints, GNBox can find parameter values of GRNs represented in Thomas' framework.

#### GINsim

GINsim [18-20] also uses a variant of Thomas' formalism. As in such a formalism, networks in GINsim have indeterminations representing asynchrony. GINsim computes the state transition graph of the GRN (presumably with forward traversal together with backtracking because of the indeterminations) before proceeding to analyze a trajectory selected by the user. GINsim can also classify circuits in the interaction diagram (i.e., can identify "functional" circuits) and can compute the set of all (stable) steady states of GRNs which do not have indeterminations using MDDs, a multi-value generalization of BDDs. Finally, GINsim can find the strongly connected components of the state-transition graph or the interaction graph.

#### SMBioNet and Mateus et al.'s system

SMBioNet [23,24] employs a variant of Thomas' formalism as well. The input is an interaction diagram of the GRN under study, together with desired properties expressed as CTL formulas. The output is a set of all the models conforming to the given interaction diagram and which also satisfy the given formulas. Candidate models are generated by instantiating parameters and then tested with a model checker.

Another system also using both Thomas' formalism and temporal logic is that by Mateus et al. [39]. Inequalities over the parameters of the model are obtained from the interaction diagram. These inequalities are augmented with LTL formulas specifying desirable properties of the model. The model is traversed forward and paths that do not satisfy the constraints are eliminated, so that only paths satisfying the constraints are retained.

#### SQUAD

SQUAD [25-27] combines a continuous model, employing ordinary differential equations, with a Boolean model of the network. The user provides the interaction diagram of the network, from which SQUAD obtains a continuous model. To find steady states of the continuous model, SQUAD first converts such a model into an approximate Boolean asynchronous model. (Thomas' formalism is not used because such a formalism has "proved to scale badly for large networks" [26].) In the Boolean model, SQUAD then computes, using BDDs and a random device, the set of states probably belonging to attractors of any size and occurring in attractors without indeterminations (called "steady" states in [26,27]). Next, SQUAD repetitively uses such states as initial states in a continuous simulator to search for steady states in the continuous model. Perturbations may be introduced to confirm that such steady states are stable and to identify the effect of specific genes.

#### GNA

GNA [40,69-72] is based on piecewise-linear differential equations. Unlike other systems using this formalism, the user need not specify precise values of parameters. Instead, less precise *intervals *are employed. States are qualitative and represent ranges of concentrations of proteins, so that simulations are also qualitative. In addition, GNA computes a discrete abstraction [73] of the continuous model, that can be verified with standard model checkers (NuSMV and CADP). The user in this case can express simple properties in CTL. For more complex properties, the GNA group has developed its own logic, called Computation Tree Regular Logic [74]. This logic extends CTL with regular expressions and fairness operators, allowing the expression of properties such as multistability and oscillations. Finally, GNA has a formula editor, guiding the user in writing new formulas.

#### BIOCHAM

BIOCHAM [41] can analyze and simulate *biochemical *networks using Boolean, kinetic, and stochastic models. In addition, properties can be formalized in temporal logic (CTL or LTL with numerical constraints), so that a model checker can be used to validate such properties. BIOCHAM models a network of protein interactions as a set of biochemical reaction rules, such as A+B = > C. Indeterminations appear because such a rule, for instance, is translated into four transitions going out of the same state, resulting from the four combinations of either reactant A or reactant B being completely or incompletely consumed. In addition, BIOCHAM has a model-update module, repairing models that do not satisfy the formalized properties.

### Comparison and planned features

On the one hand, compared with systems employing constraints, *Antelope*, by using BDDs, can compute large sets of states having a certain CTL property (e.g., a basin of attraction). On the other hand, compared with simulators, in addition to this benefit, *Antelope *can prove assertions about infinitely many paths, as opposed to only drawing statistical conclusions. It is interesting to observe, though, that some systems built around a simulator (e.g., GINsim and SQUAD) leave the simulation technique for BDDs when calculating steady states (or approximations to such states).

We also find differences between *Antelope *and other systems using model checking. For instance, SMBioNet, Mateus et al.'s system, GNA, and BIOCHAM perform model checking for *verification*, using a model checker to confirm or deny that a certain formula is satisfied. *Antelope*, by comparison, employs model checking for *calculating *sets of states.

A first clear limitation of *Antelope *when compared with systems based on Thomas' formalism (GNBox, GINsim, SMBioNet, and Mateus et al.'s system) is its being restricted to Boolean genes. We thus plan to extend *Antelope *with multi-valued genes. In this case, it would be interesting to try to incorporate into *Antelope *techniques using constraints, like those of GNBox, for determining parameter values.

Currently, *Antelope*'s GRNs are only either completely synchronous or completely asynchronous. Another improvement would then be the possibility of representing partially asynchronous GRNs, as employed in [10]. Many of the systems we reviewed allow the user to draw the GRN, whereas currently *Antelope *only accepts textual formats for describing the GRN. Clearly, future versions of *Antelope *should also have such drawing capabilities. In addition, GNA, for instance, has a formula editor, which would be desirable in *Antelope *as well. By contrast, *Antelope *is a web application, requiring no installation of any local software from the user other than a standard web browser. Moreover, *Antelope *can also run locally, exhibiting advantages of both web and local applications.

We can mention two further additions requiring more substantial work. BIOCHAM has an update module, repairing faulty models. A similar update module would also enhance *Antelope*'s features.

Another improvement, as with any model checker, would be the addition of more powerful methods for approaching the state-explosion problem. Currently, *Antelope *only has BDDs for representing large sets of states, but new techniques, such as CEGAR (Counterexample-guided abstraction refinement) [75] would enable *Antelope *to deal with larger GRNs.

## Conclusions

Systems for analyzing and building Boolean GRNs employ branching time almost exclusively for representing asynchronous transitions. Thomas' work, however, represents two other important phenomena with branching time, namely incomplete specifications and environment interaction. A consequence of including these two other kinds of indetermination is that unstable steady states may appear. We have shown how having both stable and unstable steady states is useful for developing Boolean GRNs.

In addition, we reviewed and extended the advantages of model checking, as compared with simulation, in the presence of indeterminations. In particular, we observed that model checkers, unlike simulators randomly selecting a successor, can prove properties of a set of infinitely many paths. Another advantage we reviewed is that of handling new, unforeseen properties: While model checkers can often represent new properties with additional temporal-logic formulas, simulators require the incorporation of such properties in their program code.

We illustrated the advantages of two extensions to ordinary model checking. First, we noted that ordinary model checkers would only confirm or deny that all the states in a *given *set of states have a certain property. By contrast, we claimed that model checkers are more useful for reasoning about Boolean GRN when *exhibiting *the set of states that have a property of interest. Second, we observed that the logics (e.g., CTL and LTL) underlying many model checkers are not expressive enough for representing many interesting properties of Boolean GRNs. *Antelope *tries to overcome these two limitations by showing the set of states satisfying a given formula, and by employing a hybrid extension of CTL.

It is important to remark that model checkers for hybrid logics are both relevant and neglected. As pointed out in [76], "The implementation of model checkers for hybrid logics still remains a quite unexplored field of research". Other than *Antelope*, we only know of two hybrid model checkers [52,76]. These, however, employ a basic modal logic instead of CTL, and their implementations do not use BDDs. This makes *Antelope *the first symbolic model checker for Hybrid CTL (as far as we know) with which to experiment in the development of Boolean GRNs.

## Availability and requirements

•**Project name: ***Antelope*

•**Project home page: **http://turing.iimas.unam.mx:8080/AntelopeWEB/

•**Operating system(s): **Platform independent

•**Programming language: **Java

•**Other requirements: **Any standard web browser

•**License: **GPL

•**Any restrictions to use by non-academics: **none other than those in GPL

## Authors' contributions

GA did most of the web interface. JA participated in the design of, and wrote the code for, the previous version of *Antelope*'s model checker. EA contributed to the design of the stem cell niche GRN from the literature data, used *Antelope*, and participated in writing the biology part of this paper, as well as the manual. MB also contributed to the design of the stem cell niche GRN from the literature data and wrote the rest of the biology part of this paper, as well as the manual. MC suggested using Hybrid CTL to overcome CTL limitations, participated in the design of *Antelope*, contributed to the presentation of these results, and wrote the formal definitions of CTL and Hybrid CTL (additional file 2). PG wrote the code for *Antelope*'s model checker, connected the model checker with the web interface, embedded *Antelope *and *Apache Tomcat *in a single file, did the rest of the web interface, and added numerous features to *Antelope*. DAR participated in the design of *Antelope *and wrote the model-checking part of this paper. ERAB put forward the idea of testing Kauffman's hypothesis that Boolean GRNs can recover experimental gene expression profiles, and led the translation of actual data into the the stem cell niche GRN. All authors read and approved the final manuscript.

## Supplementary Material

Additional file 1 **A gentle introduction to (Hybrid) Computation-Tree Logic**. This additional file has gentle introductions to Computation-Tree Logic and Hybrid Computation-Tree Logic. Click here for file

Additional file 2 **(Hybrid) Computation-Tree Logic**. This additional file has formal definitions of Computation-Tree Logic and Hybrid Computation-Tree Logic. Click here for file

Additional file 3 **Benchmarks**. This additional file shows the execution time for several examples. Click here for file

Additional file 4 **Antelope User's Manual**. This additional file has the *Antelope *user's manual. Click here for file
