# Supplementary material for: "Antelope": a hybrid-logic model checker for branching-time Boolean GRN analysis
Source: BMC Bioinformatics. 2011 Dec 22;12:490. doi: 10.1186/1471-2105-12-490 (PMC3316443; doi:10.1186/1471-2105-12-490)
Supplement: Additional file 1 — A gentle introduction to (Hybrid) Computation-Tree Logic. This additional file has gentle introductions to Computation-Tree Logic and Hybrid Computation-Tree Logic. [file 1471-2105-12-490-S1.PDF]

Additional file 1: A Gentle Introduction to (Hybrid) Computation-Tree Logic

David A. Rosenblueth

This additional file is an introduction both to Computation-Tree Logic (CTL) and to Hybrid CTL.

1 Computation-Tree Logic

This first section is devoted to CTL. We refer the reader to [2, 3, 5, 6, 8] for more thorough treatments; additional file 2 of this paper has a formal definition of CTL.

Example. Consider a GRN with two genes, x and y. Gene x activates itself but represses gene y, while gene y activates itself. The interaction diagram of this GRN is depicted in Fig. 1. Note that when either both x and y are active

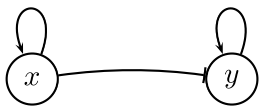

Figure 1: *The interaction diagram of a two-gene GRN. Ordinary arrow heads denote activation; T-bar arrow heads denote inhibition. Hence, gene x activates itself but represses gene y, while gene y activates itself. In case both x and y are active, we do not know whether y will be active or inactive in the next state.*

or both are inactive the next value of y is not determined by the interaction diagram in Fig. 1. The reason is that we would have to assume which of the two delays of the interactions on y is smaller or which of the two intensities is stronger. Hence, there are *indeterminations* in the next value of y.

We obtain a behavior specification as follows. The Boolean functions defining this GRN's behavior become Boolean *relations* as a result of indeterminations.

x	x'
0	0
1	1

x	y	y'
0	0	$*$
0	1	1
1	0	0
1	1	$*$

The columns labeled with unprimed letters denote the current gene values and those labeled with primed letters denote the gene values at the next time step. The star denotes indetermination.

The state-transition graph of this GRN can be viewed as a Kripke structure having the graphical representation in Fig. 2. Observe that each state is labeled

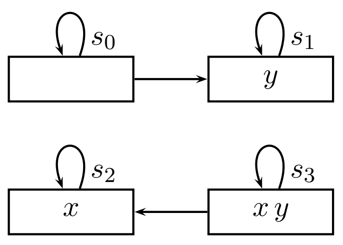

Figure 2: *A Kripke structure exemplifying branching time: both state s_0 and state s_3 have two possible immediate future states. In this case, we use branching time to model the fact that we do not know which effect will be stronger: the repressing activity of x on y or the activation activity of y on y. The interaction diagram of the GRN of this Kripke structure appears in Fig. 1.*

with the set of genes which are active in that state. State s_0 is labeled with the empty set of Boolean variables, s_1 with $\{y\}$, s_2 with $\{x\}$, and s_3 with $\{x, y\}$.

Paths. A *path* in a Kripke structure is an infinite sequence of states such that every two consecutive states are linked by a transition (i.e., an arrow or an "iteration"). For example, $(s_0, s_1, s_1, s_1, \ldots)$ is a path that starts in s_0.

Boolean formulas. Because x labels s_2 and s_3, we say that the formula "x" is true (or holds) in s_2 and in s_3. By contrast, x does not label s_0 or s_1 and therefore "x" does not hold at s_0 or s_1.

Similarly, the formula "x **or** y" holds at s_1, s_2, and s_3, because all these states are labeled with either x or y, but not so at s_0. The formula "(**not** x) **and** y", in turn, only holds at s_1.

Temporal operators. CTL has also "temporal operators", allowing us to refer to formulas holding in the future of a particular state. In this case, we must indicate whether we mean some future or all futures. Hence, it is possible to refer either (1) to *some* path starting in the present with the "modality" **E**, or (2) to *all* paths starting in the present with the modality **A**, Similarly, it is possible to refer (a) to the immediate future with the modality **X**, (b) to any state in the present or any point in the future with the modality **F**, or (c) to all states in the present and in the future with the modality **G**. The following table summarizes these modalities.

modality	*meaning*
**E**	some path (i.e., there Exists a path)
**A**	All paths
**X**	neXt state (i.e., immediate future)
**F**	any state either in the present or in the Future
**G**	all states in the present and in the future (Global)

A *temporal operator* is composed of a modality in the upper part together with a modality in the lower part of this table, which results in six temporal operators. (Often more temporal operators are included in CTL; see [8], for example.)

Consider first the modality **X**. The formula "**EX** ((**not** x) **and** y)", for instance, holds at s_0. The reason is that "(**not** x) **and** y" holds at s_1, which is in the immediate future of s_0. Similarly, "**AX not** x" holds at s_0 because "**not** x" holds at all states (s_0 and s_1) in the immediate future of s_0. By contrast, "**AX** ((**not** x) **and** y)" does not hold at s_0.

Take now the modality **F**. The formula "**EF** ((**not** x) **and** y)" holds at s_0 because there exists a path $(s_0, s_1, s_1, s_1, \ldots)$ at which "(**not** x) **and** y" holds either in the present or in the future (in this case the future). In turn, the formula "**AF** ((**not** x) **and**(**not** y))" also holds at s_0 because for all paths starting in s_0, "(**not** x) **and**(**not** y)" holds either in the present or in the future (in this case the present).

Finally, we exemplify the modality **G**. The formula "**EG** (**not** y)" holds at s_0 because "**not** y" holds at all states of some path starting in s_0, namely

$(s_0, s_0, s_0, s_0, \ldots)$. A formula holding at all states of all paths starting in s_0 would be "**not** x". Therefore, "**AG** (**not** x)" holds at s_0.

Model checkers: one state vs. all states. When the formula or the model are large, it is convenient to use a model checker. The simplest kind of model checker determines whether or not a formula holds at a particular state. It is often more useful, however, to have a model checker solving the more general problem of calculating the set of *all* states satisfying a formula. We can think of this more general model checker as *enumerating* (i.e., listing) all states and successively verifying, with the simpler kind of model checker, whether or not the given formula holds at each state, one by one.

State-identifying formulas and some properties expressible in CTL. State s_1 in Fig. 2 can be identified with the formula "(**not** x) **and** y" (i.e., this formula is true only at s_1). *We can identify any particular state with the conjunction of the names of all active genes together with the negation of all inactive genes in such a state.* State-identifying formulas are useful, for instance, for computing basins of attraction. The formula "**EF** ((**not** x) **and** y)" can be used, for example, to characterize the basin of attraction of s_1 with a model checker computing all states at which a given formula holds. This formula is true exactly at those states from which it is possible to reach s_1, namely s_1 and s_0.

Other CTL formulas can characterize, for instance, those states in the basin of attraction of a given state s that necessarily reach another state s' before reaching s. See [4] for a list of CTL formulas specifying various biological properties.

Some properties not expressible in CTL. By contrast, there does not exist a CTL formula for characterizing steady states (i.e., a formula holding exactly at the set of all steady states of an arbitrary Boolean GRN) [4]. Similarly, CTL cannot specify the states from which it is possible for a gene to oscillate (i.e., a gene which switches infinitely many times back and forth between 0 and 1). To be sure, there exists a CTL formula [4], namely "**EG** (($x \rightarrow$ **EF not** x) **and**(**not** $x \rightarrow$ **EF** x))", where "$A \rightarrow B$" abbreviates "**not** A **or** B", approximating oscillations. This formula is necessary but not sufficient for oscillations, thus possibly producing false positives but no false negatives (i.e., such a formula holds at all states from which there are oscillations, but may also hold at some states from which there are no oscillations). An example of a Kripke structure and a state satisfying this formula without having oscilla-

tions are the Kripke structure in Fig. 2 and state s_0. (Another state in this situation is s_3.)

2 Hybrid Computation-Tree Logic

This second section is devoted to Hybrid CTL. We refer the reader to [1, 7] for more thorough treatments of hybrid logics; additional file 2 of this paper has a formal definition of Hybrid CTL.

2.1 Intuition behind state variables

Next, we give an intuitive justification for extending CTL with "state" variables. We have observed that several interesting genetic properties cannot be expressed in CTL, such as state stability [4]. Let us extend CTL so as to be able to express properties such as this one.

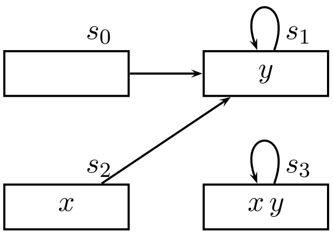

Figure 3: *A Kripke structure for illustrating a formula characterizing steady states.*

A first step in extending the "steady-state" formula to an arbitrary state. CTL does have a formula holding when a *particular* state is steady. Consider, for example, the Kripke structure in Fig. 3, where s_1 is a steady state. A formula identifying s_1 (i.e., a formula holding only at s_1) is "$(\textbf{not}\,x)\,\textbf{and}\,y$". If we precede this formula by $\mathbf{AX}$, we obtain "$\mathbf{AX}\,((\textbf{not}\,x)\,\textbf{and}\,y)$", which holds at s_1 (and possibly at other states as well, such as s_0 and s_2). By contrast, take now s_2, which is not a steady state. A formula identifying s_2 is "$x\,\textbf{and}(\textbf{not}\,y)$". Preceding this formula by $\mathbf{AX}$, we get "$\mathbf{AX}\,(x\,\textbf{and}(\textbf{not}\,y))$", which does not hold at s_2 (although such a formula may hold at other states). Every formula starting with $\mathbf{AX}$ and followed by a subformula identifying a state holds at such a state if such a state is steady.

Hence, to extend CTL so as to be able to have a formula holding exactly in all steady states, we need a mechanism for varying the subformula following **AX**, identifying a particular state, through all the states.

Implicit enumeration of states. Recall now that we can conceptually think of the calculation of the set of all the states at which a CTL formula holds as successively enumerating all states, and determining whether or not such a formula holds at each state. Hence, the needed enumeration is already performed by the CTL model checker. The hindrance for characterizing an arbitrary steady state is rather that the formula does not have access to the "current" state in such an enumeration. This suggests extending formulas with "state" variables which take as value the current state.

State variables as a means to access the current state. The hybrid extension of CTL formulas essentially consists in such an addition of state variables. We will use σ for one such variable. If σ successively takes as value the formulas identifying the different states (i.e., "$(\mathbf{not}\, x)\, \mathbf{and} (\mathbf{not}\, y)$", "$(\mathbf{not}\, x)\, \mathbf{and}\, y$", "$x\, \mathbf{and} (\mathbf{not}\, y)$", and "$x\, \mathbf{and}\, y$" in our example), then the formula "$\mathbf{AX}\, \sigma$" will hold exactly at all steady states (i.e., s_1 and s_3 in our example). In hybrid logic, however, we must explicitly indicate that σ is to be set to the current state with the "$\downarrow$" operator. The full formula would be: "$\downarrow\sigma.\mathbf{AX}\, \sigma$".

By definition, the formula "$\downarrow\sigma.\mathbf{AX}\, \sigma$" holds at a state s if and only if the formula obtained from "$\mathbf{AX}\, \sigma$" after replacing σ by the subformula identifying s (in our example we would have: "$(\mathbf{not}\, x)\, \mathbf{and} (\mathbf{not}\, y)$" for s_0, "$(\mathbf{not}\, x)\, \mathbf{and}\, y$" for s_1 "$x\, \mathbf{and} (\mathbf{not}\, y)$" for s_2, and "$x\, \mathbf{and}\, y$" for s_3, respectively), holds at s.

Intuitively, then, "$\downarrow\sigma.\mathbf{AX}\, \sigma$" holds exactly at all states whose only outgoing transition is a self-loop. Similarly, "$\downarrow\sigma.\mathbf{EX}\, \sigma$" holds exactly at all states which have a self-loop, and possibly other outgoing transitions as well.

2.2 Other formulas

Attractors of various sizes. The notion of a steady state can be generalized in an *attractor*, involving more than one state. A steady state would then be a one-state attractor. A formula characterizing attractors of any size would be: "$\downarrow\sigma.\mathbf{EX}\, \mathbf{EF}\, \sigma$", which can be understood as follows. Note first that the modality "$\mathbf{EX}\, \mathbf{EF}$" denotes a *strict* future (i.e., "$\mathbf{EF}$" denotes either the present or the future and "$\mathbf{EX}$" denotes the next state). Hence, "$\downarrow\sigma.\mathbf{EX}\, \mathbf{EF}\, \sigma$" holds at a state s if and only if the formula obtained from "$\mathbf{EX}\, \mathbf{EF}\, \sigma$" after replacing σ by the subformula identifying s holds at s. Therefore, "$\downarrow\sigma.\mathbf{EX}\, \mathbf{EF}\, \sigma$"

holds at all states occurring in the future of themselves, i.e., attractors of any size.

Another interesting formula would be "$\downarrow\sigma.$**EX** ((**not** σ) **and EX** σ)", which holds at states belonging to an attractor of size two. We can think of this formula as follows: The current state, σ, has a successor which is not σ, which in turn has σ as a successor. We refer the reader to the *Antelope* web site (`http://turing.iimas.unam.mx:8080/AntelopeWEB/`) for formulas holding at different types of cycles.

A formula for possible oscillations. The basin of attraction of an attractor in which a gene x oscillates can be characterized with the formula "**EF** $\downarrow\sigma.$**EF** ((**not** x) **and EF** (x **and** σ))". This formula can be understood as follows. The rightmost subformula "x **and** σ" establishes that σ is a state at which "x" holds. Considering now a larger subformula, let τ be any state at which "(**not** x) **and EF** (x **and** σ)" holds. Intuitively, such a formula expresses that gene x oscillates: The subformula "**not** x" establishes that "x" does not hold at τ, while the subformula "**EF** (x **and** σ)" means that σ can be found in the future from τ (and that "x" holds at σ). Next, "$\downarrow\sigma.$**EF** ((**not** x) **and EF** (x **and** σ))" holds in turn if τ can be found in the future from σ. Finally, "**EF** $\downarrow\sigma.$**EF** ((**not** x) **and EF** (x **and** σ))" denotes the basin of attraction of σ.

Computation of the set of states satisfying EX obtains all previous states. Consider again the example in Fig. 3. Observe that the formula "**EX** ((**not** x) **and** y)" holds at all states which have a *next* state at which "(**not** x) **and** y" holds. The formula "(**not** x) **and** y", in turn, holds at s_1. Hence, computing the states at which "**EX** ((**not** x) **and** y)" holds results in going *backwards* in time one step, obtaining s_0 and s_2.

Computation of the set of states satisfying EY obtains all next states. There exists an operator having the inverse effect to that of **EX**, called **EY**, for "Exists" and "Yesterday". **EY** φ holds at all states which have an *immediately previous* state at which φ holds. In our example in Fig. 3, "(**not** x) **and**(**not** y)" holds at s_0. Remark that s_1 has an immediately previous state at which "(**not** x) **and**(**not** y)" holds (s_0). Consequently, computing the states at which "**EY** ((**not** x) **and**(**not** y))" holds results in going *forward* in time one step, obtaining s_1.

Thorough treatments. Often more operators are included in hybrid logics. We refer the reader for instance to [1, 7] for deeper treatments of hybrid logics.

References

[1] Carlos Areces and Balder ten Cate. Hybrid logics. In P. Blackburn, F. Wolter, and J. van Benthem, editors, *Handbook of Modal Logics*, pages 821–868. Elsevier, 2006.

[2] Christel Baier and Joost-Pieter Katoen. *Principles of Model Checking.* MIT Press, 2008.

[3] B. Bérard, M. Bidoit, A. Finkel, F. Laroussinie, A. Petit, L. Petrucci, Ph. Schnoebelen, and P. McKenzie. *Systems and Software Verification. Model-Checking Techniques and Tools.* Springer, 2001.

[4] Nathalie Chabrier-Rivier, Marc Chiaverini, Vincent Danos, François Fages, and Vincent Schächter. Modeling and querying biomolecular interaction networks. *Theoretical Computer Science*, 325:25–44, 2004.

[5] E. M. Clarke, E. A. Emerson, and A. P. Sistla. Automatic verification of finite-state concurrent systems using temporal logic specifications. *ACM Transactions of Programming Languages and Systems*, 8(2):244–263, 1986.

[6] Edmund M. Clarke, Orna Grumberg, and Doron A. Peled. *Model Checking.* MIT Press, 1999.

[7] Massimo Franceschet and Maarten de Rijke. Model checking hybrid logic (with an application to semistructured data). *Journal of Applied Logic*, 4(2):168–191, 2006.

[8] Michael R. A. Huth and Mark D. Ryan. *Logic in Computer Science: Modelling and reasoning about systems.* Cambridge University Press, 2nd edition, 2004.
