# Supplementary material for: "Antelope": a hybrid-logic model checker for branching-time Boolean GRN analysis
Source: BMC Bioinformatics. 2011 Dec 22;12:490. doi: 10.1186/1471-2105-12-490 (PMC3316443; doi:10.1186/1471-2105-12-490)
Supplement: Additional file 2 — (Hybrid) Computation-Tree Logic. This additional file has formal definitions of Computation-Tree Logic and Hybrid Computation-Tree Logic. [file 1471-2105-12-490-S2.PDF]

Additional file 2: (Hybrid) Computation-Tree Logic

Miguel Carrillo

In this additional file, we present a version of the Computation-Tree Logic (*CTL*) [1, 3] and a version of the Hybrid Computation-Tree Logic (*HCTL*) [2]. First, we introduce models for these two logics. Then, we present the syntax of *CTL* and *HCTL*. Finally, we give a semantics for *HCTL* including that of *CTL*.

The basic notation that we use in this additional file is as follows. If D is a set, we use $\mathcal{P}(D)$ for the power set of D, and we use $\sharp(D)$ to denote the cardinality of D. If $R \subseteq D \times D$ is a binary relation, then we say that R is *serial* iff $\forall x \in D$, $\exists y \in D$ such that $(x, y) \in R$. We use xRy to denote that $(x, y) \in R$.

1 (Hybrid) Kripke models

The semantics of *CTL* and *HCTL* respectively need Kripke models and hybrid Kripke models. So we include below a definition Kripke model that can be extended to a definition of hybrid Kripke model.

We assume that AP is a nonempty finite set and we call the elements of AP *atomic propositions*. Besides, we consider a fixed subset of AP, $N \subseteq AP$, and we call the elements of N *nominals*.

Definition 1.1 (Kripke model)**.** *We say that M is a* finite Kripke model *for AP iff $M = (S, R, L)$ where*

1. *$S \neq \emptyset$ is a finite set.*
2. *$R \subseteq S \times S$ is a serial relation.*
3. *$L : AP \to \mathcal{P}(S)$.*

We call the elements of S states. *R is the* accessibility relation *of M, and we call the elements of R* transitions. *We refer to L as the* labeling function *of M. If $s \in L(p)$, we say that p is a* label *of s. Intuitively, $L(p)$ is the set of states where p is true.*

Since we assume that the accessibility relation of a model is serial, no state of the model can deadlock. This requirement is a technical convenience, and does not represent any restriction on the modeling ability of Kripke models (e.g. [3, p. 178]). If we need to model a system having deadlock states, we could add to the Kripke model a state s_d representing deadlock, and transitions sRs_d for each deadlock state s, as well as the transition s_dRs_d.

The above definition of labeling function in a Kripke model deviates from the standard definition where $L : S \to \mathcal{P}(AP)$. The definition above, equivalent to the standard definition, is suitable for the treatment of nominals.

To extend the definition of Kripke model to that of hybrid Kripke model, we have to consider the labeling by nominals as a special case:

Definition 1.2 (Hybrid Kripke model)**.** *We say that M is a* finite hybrid Kripke model *for AP and N iff $M = (S, R, L)$, where*

1. *$S \neq \emptyset$ is a finite set,*
2. *$R \subseteq S \times S$ is a serial relation, and*
3. *$L : AP \to \mathcal{P}(S)$ is such that $\forall n \in N.\ \sharp(L(n)) = 1$*

Since $n \in N$ implies that there is $s \in S$ such that $L(n) = \{s\}$, we say that state s is named by *n. Therefore, nominals are true at exactly one state in any hybrid Kripke model. Thus, a state named by a nominal may be mentioned in a formula by using such a nominal.*

If M is a (hybrid) Kripke model, when necessary we use a superscript M to denote its components, i.e. $M = (S^M, R^M, L^M)$.

To define the semantics of hybrid operators we will need to modify the labeling by nominals in a model. We make these modifications by forcing a state to be named by a nominal.

Definition 1.3 (Name change)**.** *Let M be a hybrid Kripke model, $n \in N$, and $s \in S^M$.*

1. *We define the labeling function $L^M[n \mapsto s] : AP \to \mathcal{P}(S^M)$, L^M* with *s* named by *n, by: $L^M[n \mapsto s](n) = \{s\}$, and for all $p \in AP$ such that $p \neq n$, $L^M[n \mapsto s](p) = L^M(p)$.*

2. *We define the hybrid Kripke model* $M[n \mapsto s]$*,* M with s named by n*, by:* $S^{M[n\mapsto s]} = S^M$*,* $R^{M[n\mapsto s]} = R^M$*, and* $L^{M[n\mapsto s]} = L^M[n \mapsto s]$.

A *path* in a (hybrid) Kripke model M is an infinite sequence of states, $\pi\colon \mathbb{N} \to S^M$, such that for all $i \in \mathbb{N}$, $(\pi_i, \pi_{i+1}) \in R^M$; as usual we use π_i instead of $\pi(i)$. The *set of paths in* M *starting at* s is $\Pi_{M,s} = \{\pi \mid \pi \text{ is a path in } M \text{ and } \pi_0 = s\}$.

2 *CTL* and *HCTL* syntax

We present here a syntax for *CTL*. Then we extend this syntax to get a syntax for *HCTL*.

We assume that *CTL* formulas, Φ, have the following syntax in Backus-Naur form (BNF):

$$\Phi ::= \top \mid p \mid (\neg\Phi) \mid (\Phi \wedge \Phi) \mid (\mathbf{EX}\,\Phi) \mid \mathbf{E}[\Phi\ \mathbf{U}\ \Phi] \mid (\mathbf{AF}\,\Phi)$$

where p ranges over the set of atomic propositions AP.

The propositional operators $\top$, $\neg$, and $\wedge$ have the usual meaning: truth, negation and conjunction, respectively. Intuitively, **E** and **A** respectively stand for "there exists a path" and "for all paths". For the temporal operators we have that **X** means "at a next state", **U** "until", and **F** stands for "either now or in the future".

We can define other operators in terms of that considered above. First, we define other propositional operators, $\perp$ (falsity), $\vee$(disjunction), $\Rightarrow$(implication) and $\Leftrightarrow$(equivalence):

$$\perp = \neg\top \qquad\qquad \alpha \vee \beta = \neg(\neg\alpha \wedge \neg\beta)$$
$$\alpha \Rightarrow \beta = \neg\alpha \vee \beta \qquad\qquad \alpha \Leftrightarrow \beta = (\alpha \Rightarrow \beta) \wedge (\beta \Rightarrow \alpha)$$

Second, we define the operators **AX** and **EG**:

$$\mathbf{AX}\,\alpha = \neg\mathbf{EX}\,\neg\alpha \qquad\qquad \mathbf{EG}\,\alpha = \neg\mathbf{AF}\,\neg\alpha$$

Then, operator **AU** has a special definition (e.g. see [3, p. 216]):

$$\mathbf{A}[\alpha\,\mathbf{U}\,\beta] = \neg(\mathbf{E}[\neg\beta\,\mathbf{U}\,(\neg\alpha \wedge \neg\beta)] \vee \mathbf{EG}\,\neg\beta)$$

Finally, we define other *CTL* operators:

$$\mathbf{EF}\,\alpha = \mathbf{E}[\top\,\mathbf{U}\,\alpha] \qquad \mathbf{AG}\,\alpha = \neg\mathbf{EF}\,\neg\alpha$$
$$\mathbf{E}[\alpha\,\mathbf{R}\,\beta] = \neg\mathbf{A}[\neg\alpha\,\mathbf{U}\,\neg\beta] \qquad \mathbf{A}[\alpha\,\mathbf{R}\,\beta] = \neg\mathbf{E}[\neg\alpha\,\mathbf{U}\,\neg\beta]$$

Intuitively, **G** stands for "always" or "globally", and **R** means "release".

Hybrid *CTL*, denoted by *HCTL*, is an extension of *CTL* that considers three *hybrid operators*: the *at operator*($@n$), the *down-arrow binder*($\downarrow n$), and the *existential binder*($\exists n$).

The syntax in BNF for *HCTL* formulas (Ψ) is as follows:

$$\Psi ::= \top \mid p \mid (\neg\Psi) \mid (\Psi \wedge \Psi) \mid (\mathbf{EX}\,\Psi) \mid \mathbf{E}[\Psi\ \mathbf{U}\ \Psi] \mid (\mathbf{AF}\,\Psi) \mid$$
$$(@n.\Psi) \mid (\downarrow n.\Psi) \mid (\exists n.\Psi)$$

where p ranges over AP, and n ranges over the set of nominals N.

Intuitively, $@n$ means "at the state named by n", $\downarrow n$ stands for "bind n to the current state", and $\exists n$ means "(non-deterministically) bind n to some state".

3 *CTL* and *HCTL* semantics

We now proceed to define the semantics of *CTL*. Then, we extend this semantics to a semantics of *HCTL*.

Definition 3.1 (*CTL* semantics)**.** *Let M be a Kripke model, $s \in S^M$, and $\varphi \in CTL$. We define $(M,s) \models \varphi$, M* satisfies *φ* at *s, recursively on the structure of φ:*

1. $(M,s) \models \top$
2. $(M,s) \models p$ *iff* $s \in L^M(p)$
3. $(M,s) \models \neg\alpha$ *iff* $(M,s) \not\models \alpha$
4. $(M,s) \models \alpha \wedge \beta$ *iff* $(M,s) \models \alpha$ *and* $(M,s) \models \beta$
5. $(M,s) \models \mathbf{EX}\,\alpha$ *iff* $\exists\pi \in \Pi_{M,s}$ *such that* $(M,\pi_1) \models \alpha$
6. $(M,s) \models \mathbf{E}[\alpha\ \mathbf{U}\ \beta]$ *iff* $\exists\pi \in \Pi_{M,s}$ *and* $\exists i \in \mathbb{N}$ *such that:* $(M,\pi_i) \models \beta$ *and* $\forall j < i$ $(M,\pi_j) \models \alpha$
7. $(M,s) \models \mathbf{AF}\,\alpha$ *iff* $\forall\pi \in \Pi_{M,s}$, $\exists i \in \mathbb{N}$ *such that* $(M,\pi_i) \models \alpha$

Now, we get the semantics of *HCTL* by extending the semantics of *CTL* to hybrid Kripke models.

Definition 3.2 (*HCTL* semantics)**.** *Let M be a hybrid Kripke model, $s \in S^M$, and $\varphi \in HCTL$. We define $(M,s) \models \varphi$, M* satisfies φ at s*, recursively on the structure of φ:*

1. $(M,s) \models \top$
2. $(M,s) \models p$ *iff* $s \in L^M(p)$
3. $(M,s) \models \neg\alpha$ *iff* $(M,s) \not\models \alpha$
4. $(M,s) \models \alpha \wedge \beta$ *iff* $(M,s) \models \alpha$ *and* $(M,s) \models \beta$
5. $(M,s) \models \mathbf{EX}\,\alpha$ *iff* $\exists \pi \in \Pi_{M,s}$ *such that* $(M,\pi_1) \models \alpha$
6. $(M,s) \models \mathbf{E}[\alpha\ \mathbf{U}\ \beta]$ *iff* $\exists \pi \in \Pi_{M,s}$ *and* $\exists i \in \mathbb{N}$ *such that:* $(M,\pi_i) \models \beta$ *and* $\forall j < i\ (M,\pi_j) \models \alpha$
7. $(M,s) \models \mathbf{AF}\,\alpha$ *iff* $\forall \pi \in \Pi_{M,s}$, $\exists i \in \mathbb{N}$ *such that* $(M,\pi_i) \models \alpha$
8. $(M,s) \models @n.\alpha$ *iff* $L^M(n) = \{s'\}$ *and* $(M,s') \models \alpha$
9. $(M,s) \models \downarrow n.\alpha$ *iff* $(M[n \mapsto s],s) \models \alpha$
10. $(M,s) \models \exists n.\alpha$ *iff* $\exists s' \in S^M$ *such that* $(M[n \mapsto s'],s) \models \alpha$

4 *HCTL* denotational semantics

We define here an appropriate *HCTL* semantics for the implementation of a model checker. To simplify the definition the denotational semantics of *HCTL*, we will need the existential and universal preimage of a set of states as well as a mechanism of definition by fixed points.

Definition 4.1 ($Pre_\exists(X)$, $Pre_\forall(X)$)**.** *Let M is a (hybrid) Kripke model and $X \subseteq S^M$. The* existential preimage *of X in M is*

$$Pre_\exists(X) = \{s \in S^M \mid \exists s'.\ sR^M s' \text{ and } s' \in X\}.$$

The universal preimage *of X in M is analogously defined by*

$$Pre_\forall(X) = \{s \in S^M \mid \forall s'.\ sR^M s' \text{ implies } s' \in X\}.$$

Note that $Pre_\forall(X) = S^M - Pre_\exists(S^M - X)$.

Definition 4.2 (Fixed points and monotone functions). *Let D be a set, $X \subseteq D$, and $F : \mathcal{P}(D) \to \mathcal{P}(D)$ a function. We say that*

1. *X is a* least fixed point *of F iff $F(X) = X$ and, for all $Y \in \mathcal{P}(D)$, $F(Y) = Y \Rightarrow X \subseteq Y$*

2. *X is a* greatest fixed point *of F iff $F(X) = X$ and, for all $Y \in \mathcal{P}(D)$, $F(Y) = Y \Rightarrow X \supseteq Y$*

3. *F is* monotone *iff $X \subseteq Y$ implies $F(X) \subseteq F(Y)$ for all $X, Y \in \mathcal{P}(D)$.*

Note that least (greatest) fixed points are unique. Thus, we use $\mathrm{lfp}(F)$ *and* $\mathrm{gfp}(F)$ *to respectively denote the least and the greatest fixed point of F. Observe that $Pre_\exists$ and $Pre_\forall$ are monotone functions.*

If $F : \mathcal{P}(D) \to \mathcal{P}(D)$ is a function, we use $F^i(X)$ to denote that F should be applied i times: $F^1(X) = F(X)$ and $F^{i+1}(X) = F(F^i(X))$.

The following fact is a special case of a fundamental result cited as the Knaster–Tarski Theorem.

Theorem 4.1 (Existence of fixed points). *Let D be a finite set such that $\sharp(D) = n + 1$. If $F : \mathcal{P}(D) \to \mathcal{P}(D)$ is a monotone function, then* $\mathrm{lfp}(F) = F^{n+1}(\emptyset)$ *and* $\mathrm{gfp}(F) = F^{n+1}(D)$.

Proof. e.g. see [3, p. 241]. □

Definition 4.3 (*HCTL* denotational semantics). *Let M be a hybrid Kripke model, and $\varphi \in HCTL$. We define the meaning of φ in M, $[\![\varphi]\!]_M$, recursively on the structure of φ:*

1. $[\![\top]\!]_M = S^M$

2. $[\![p]\!]_M = L^M(p)$

3. $[\![\neg\alpha]\!]_M = S^M - [\![\alpha]\!]_M$

4. $[\![\alpha \wedge \beta]\!]_M = [\![\alpha]\!]_M \cap [\![\beta]\!]_M$

5. $[\![\mathbf{EX}\,\alpha]\!]_M = \{s \in S^M \mid \exists s'.\ sR^M s' \text{ and } s' \in [\![\alpha]\!]_M\} = Pre_\exists([\![\alpha]\!]_M)$

6. $[\![\mathbf{E}[\alpha\ \mathbf{U}\ \beta]]\!]_M = \mathrm{lfp}(F)$, *where*
   $F : \mathcal{P}(S^M) \to \mathcal{P}(S^M)$ *and* $F(X) = [\![\beta]\!]_M \cup ([\![\alpha]\!]_M \cap Pre_\exists(X))$

7. $[\![\mathbf{AF}\,\alpha]\!]_M = \mathrm{lfp}(G)$, *where*
   $G : \mathcal{P}(S^M) \to \mathcal{P}(S^M)$ *and* $G(X) = [\![\alpha]\!]_M \cup Pre_\forall(X)$

8. $[\![@n.\alpha]\!]_M = \begin{cases} S \textit{ if } L^M(n) \cap [\![\alpha]\!]_M \neq \emptyset \\ \emptyset \textit{ otherwise} \end{cases}$

9. $[\![\downarrow n.\alpha]\!]_M = \{s \in S^M \mid s \in [\![\alpha]\!]_{M[n \mapsto s]}\} = \bigcup_{s \in S^M}(\{s\} \cap [\![\alpha]\!]_{M[n \mapsto s]})\}$

10. $[\![\exists n.\alpha]\!]_M = \{s \in S^M \mid \exists s' \in S^M.\ s \in [\![\alpha]\!]_{M[n \mapsto s']}\} = \bigcup_{s' \in S^M}([\![\alpha]\!]_{M[n \mapsto s']})$

Note that above functions F and G are monotone. Therefore the least fixed point of both functions exists.

In our model checker, *Antelope*, operators $\top$, $\bot$, $\neg$, $\wedge$, $\mathbf{E}[\alpha\,\mathbf{U}\,\beta]$, @, $\downarrow$, and $\exists$ are respectively transliterated to `true`, `false`, `~`, `&`, `E(`α `U` β`)`, `@`, `!`, and `]`.

References

[1] Clarke EM, Emerson EA, *Design and synthesis of synchronization skeletons using branching time temporal logic.* In Proc. Workshop on Logics of Programs, IBM Watson Research Center 1981:52-71. [Lecture Notes in Computer Science No. 131].

[2] Huth MRA, *Abstraction and Probabilities for Hybrid Logics.* Electronic Notes in Theoretical Computer Science (ENTCS), Volume 112, 2005.

[3] Huth MRA, Ryan MD, *Logic in Computer Science: Modelling and reasoning about systems.* Cambridge University Press, 2nd edition, 2004.
