# Supplementary material for: "Antelope": a hybrid-logic model checker for branching-time Boolean GRN analysis
Source: BMC Bioinformatics. 2011 Dec 22;12:490. doi: 10.1186/1471-2105-12-490 (PMC3316443; doi:10.1186/1471-2105-12-490)
Supplement: Additional file 3 — Benchmarks. This additional file shows the execution time for several examples. [file 1471-2105-12-490-S3.PDF]

Additional file 3: Benchmarks

This additional file has some benchmarks of *Antelope*. The table below shows the average verification times for a few random GRNs without indeterminations, with respect to some properties. These figures do not include the time taken by the web browser to display the results. N denotes the number of genes and K denotes the average number of inputs of a gene in the interaction diagram. In the generation of these networks, the probability of the value of a gene being 1 (or 0) was $p = 0.5$. We have separated the time taken to build the BDD of the dynamics of the GRN from the time taken to perform the verification itself: The column named "BDD" shows the time taken to build such a BDD. These benchmarks were run on a laptop with 3 Gb RAM, a Core(TM)2 Duo CPU T6670 at 1.2 GHz, under Ubuntu 10.04.2. We used the Java implementation of JavaBDD [3] (as opposed to BuDDy [1]).

The formula

`!s.AX s`

holds exactly at all stable steady states, and can be taken to be representative of Hybrid CTL formulas involving a single traversal of all states of a GRN, such that for each state its predecessors are obtained. Next, the formula schema

$$\bigwedge_{i=0}^{n-1} g_i \leftrightarrow f_i(g_0, \ldots, g_{n-1}),$$

which we call *equational fixed point*, and abbreviate as `efp`, results in a more efficient way of computing the set of steady states for GRN without indeterminations; this method is used in [2], and can be used in *Antelope* by selecting the option "Use conjunctive equational fixed point formula". Finally, the formula

`EF` s

holds at all nodes in the basin of attraction of state s. This formula can be taken to be representative of formulas with a single CTL operator different from `AX` or `EX`. The entry "*of.*" means memory overflow.

N	K	BDD	*formula*		
			`!s.AX s`	`efp`	`EF` s
5	2.4	41 ms	4 ms	1 ms	32 ms (20 states)
5	2.8	53 ms	4 ms	1 ms	4 ms (12 states)
10	1.4	44 ms	71 ms	1 ms	48 ms (288 states)
10	2.5	29 ms	225 ms	1 ms	24 ms (84 states)
15	1.4	167 ms	568 ms	1 ms	[no steady states]
15	2.3	40 ms	863 ms	1 ms	[no steady states]
20	1.7	166 ms	35.3 s	1 ms	11 ms (786,432 states)
20	3.2	103 ms	51.8 s	2 ms	158 ms (966,592 states)
25	2.3	158 ms	*of.*	3 ms	112 ms (23,161,088 states)
25	3.3	687 ms	*of.*	4 ms	679 ms (1,124,568 states)
30	2.1	833 ms	*of.*	2 ms	[no steady states]
30	3.2	62.9 s	*of.*	6 ms	[no steady states]
35	2.2	9.2 min	*of.*	9 ms	4 ms (1 state)
35	3.2	*of.*	*of.*	20 ms	[no steady states]
40	1.8	*of.*	*of.*	7 ms	[no steady states]
40	2.7	*of.*	*of.*	13 ms	[no steady states]

Note first that times are not always greater for bigger networks. This can be explained by recalling that the size of a BDD is not necessarily directly proportional to the cardinality of the represented set.

We also remark that the instances causing overflow (*of.*) would presumably produce a result on a computer with more memory.

On the other hand, the times reported for computing stable stationary states with formulas of the form `efp` might seem surprisingly low. A reason for these low figures is that such formulas are propositional, and the BDD of the dynamics of the network is not used.

Finally, observe the time in the computation of a basin of attraction when $N = 25$ and $K = 2.3$. In this case, *Antelope* built a set with 2×10^7 states in 112 ms. on average.

References

[1] Jørn Lind-Nielsen. Buddy 2.4, 2004. `http://sourceforge.net/projects/buddy/`.

[2] Aurélien Naldi, Denis Thieffry, and Claudine Chaoiuya. Decision diagrams for the representation and analysis of logical models of regulatory networks.

In Muffy Calder and Stephen Gilmore, editors, *Proc. Computational Methods in Systems Biology (CMSB)*, pages 233–247, 2007. Lecture Notes in Computer Science No. 4695, Edinburgh, Scotland.

[3] John Whaley. JavaBDD 1.0b2, 2007. http://javabdd.sourceforge.net/.
