# Supplementary material for: "Antelope": a hybrid-logic model checker for branching-time Boolean GRN analysis
Source: BMC Bioinformatics. 2011 Dec 22;12:490. doi: 10.1186/1471-2105-12-490 (PMC3316443; doi:10.1186/1471-2105-12-490)
Supplement: Additional file 4 — Antelope User's Manual. This additional file has the Antelope user's manual. [file 1471-2105-12-490-S4.PDF]

ANTELOPE MODEL CHECKER

USER'S MANUAL

CONTACT INFORMATION
Pedro Góngora (pedro.gongora@gmail.com) and David Rosenblueth (drosenbl@servidor.unam.mx).

Table of Contents

ACCESSING ANTELOPE.. 3
ANTELOPE'S SCREENS………………………………………... 3
MAIN FUNCTIONS 3
Models…………………………………………………… 3
Selecting a preloaded model…………………………….. 3
Uploading a new model………………………………… 4
Using preloaded properties………………………………. 5
Using preloaded properties from the main screen……………….. 5
Using preloaded formulas from the formula properties screen….. 7
Saving your results………………………………………. 7
Synchrony and Asynchrony……………………………... 8
Define or edit custom properties………………………… 8
Add a new property……………………………………… 10
Remove a property……………………………………….. 11
Manipulating the results…………………………………. 12
Wildcards, basin of attraction, successors and predecessors…….. 12
MODEL FILE SYNTAX...……………………………….. 13
GENE NAMES………………………………………… 16
FORMULAS……………………………………………. 16
REFERENCES…………………………………………. 18

ACCESSING ANTELOPE

Antelope is freely accessible and can run either remotely or locally.

Remotely Antelope is available at the following web address: http://turing.iimas.unam.mx:8080/AntelopeWEB and the only software requirement is a web browser connected to the internet (No additional software needs to be installed).

Locally Antelope is downloadale from the same web address and the only requirement is Java version 1.6 or higher. Starting Antelope locally takes approximately 33 seconds in a computer with 3GB in RAM.

ANTELOPE'S SCREENS

Antelope has four different screens: (1) The main screen, (2) the results screen, (3) the formula properties screen and (4) the edit and create properties screen. We will now explain each screen.

MAIN FUNCTIONS

1. Models

Before you can work and analyze a model with Antelope, you must select a model to work with. Antelope has two options. You can use a number preloaded models. Among these models there are two important ones: flower GRN [1] and root GRN [2], both for the model plant *Arabidopsis*. You can alternatively upload your own model. These options are in the main screen (see figure 1).

1.1 Selecting a preloaded model

The preloaded models have been published before, so more details about these models are available in Espinosa-Soto and collaborators (2004) [1] and in Azpeitia and collaborators (2010) [2]. If you are not interested in these particular models you can still use them to test Antelope. Figure 1 shows the location of the option "Choose a gene regulatory network" on the main screen. To choose one of the preloaded models, click on the drop-down list and select a model. Once you have chosen a model you can begin analyzing it by clicking submit.

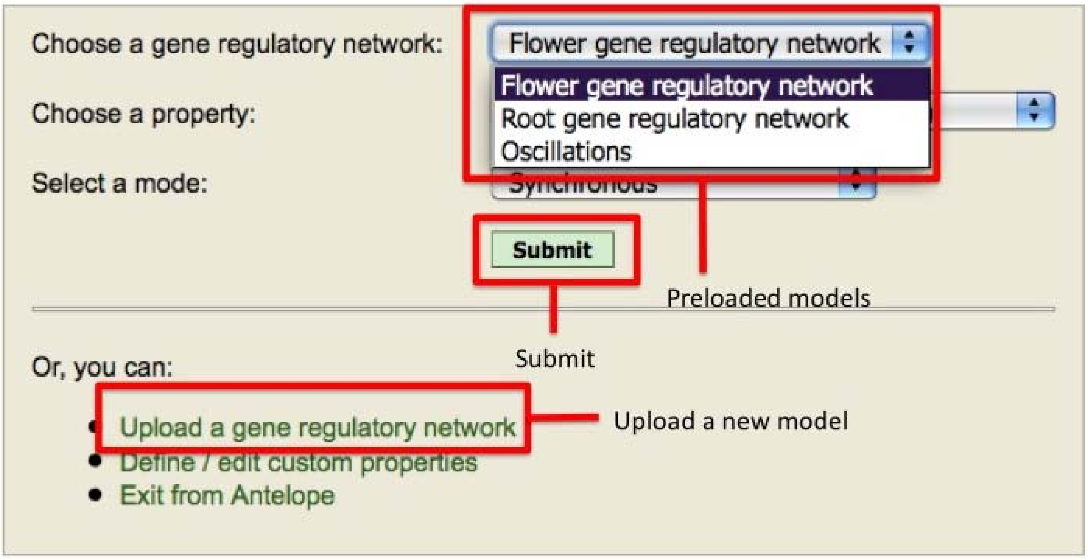

Figure 1. Selecting a preloaded model. The drop-down list with preloaded models is framed in red.

1.2 Uploading a new model

In Antelope models are stored in plain text files. See Section "Format of Models" for details.

To upload a new model click on the "Upload a gene regulatory network" option shown in figure 1. The screen changes to the one in figure 2, where you can choose a model from your hard disk.

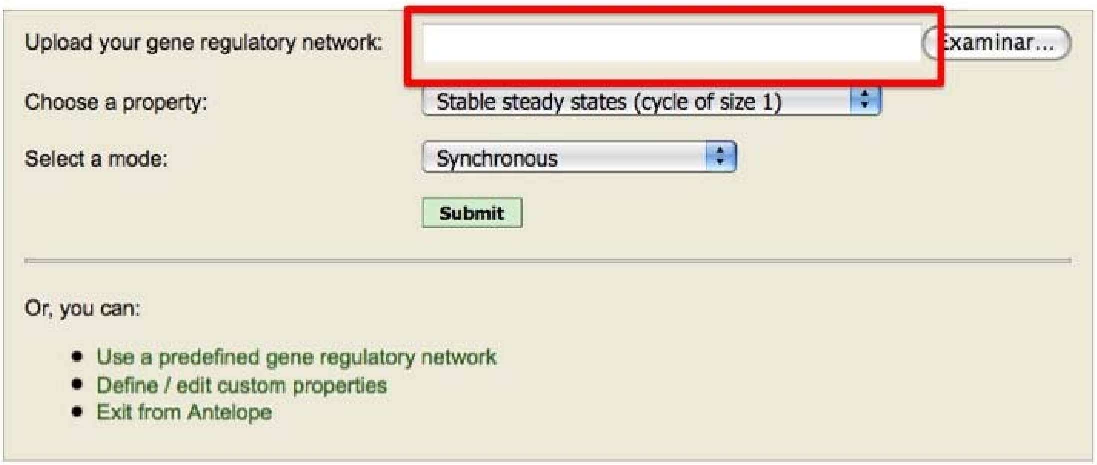

Figure 2. Upload a file. The window for the new model is framed in red.

Once you have selected the file to be loaded, the screen will change to the one shown in figure 3.

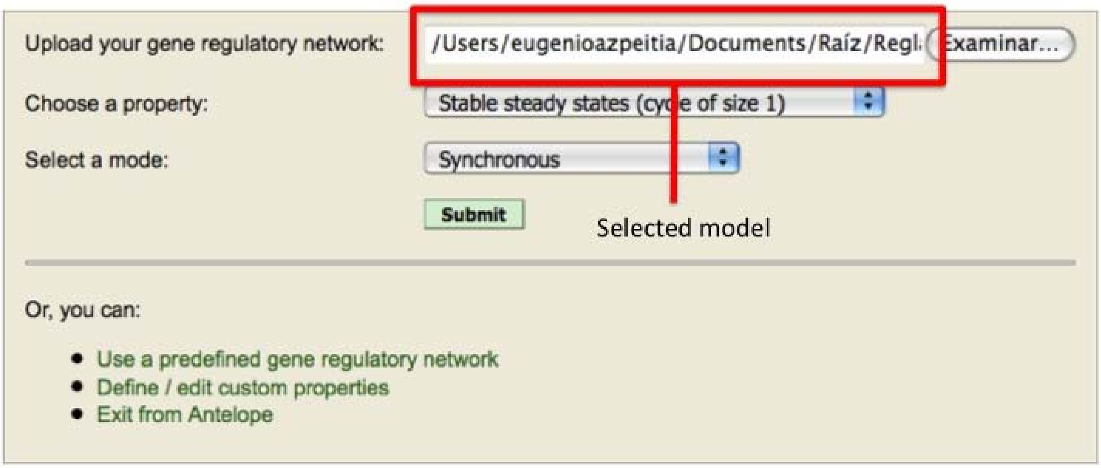

Figure 3. New model selected. The new model is framed in red.

2. Using preloaded properties

We have preloaded some formulas for the fundamental analysis of models. Examples are stable steady states, unstable steady states and cycles of various sizes.

The preloaded formulas can be used from (i) the main screen, (ii) the results screen and (iii) the formula properties screen.

2.1. Using preloaded properties from the main screen

To use a preloaded property from the main screen choose it from the drop-down list “Choose a property” (see figure 4). The information about the formulas is in the formula properties screen; see section “Edit or custom properties” for details.

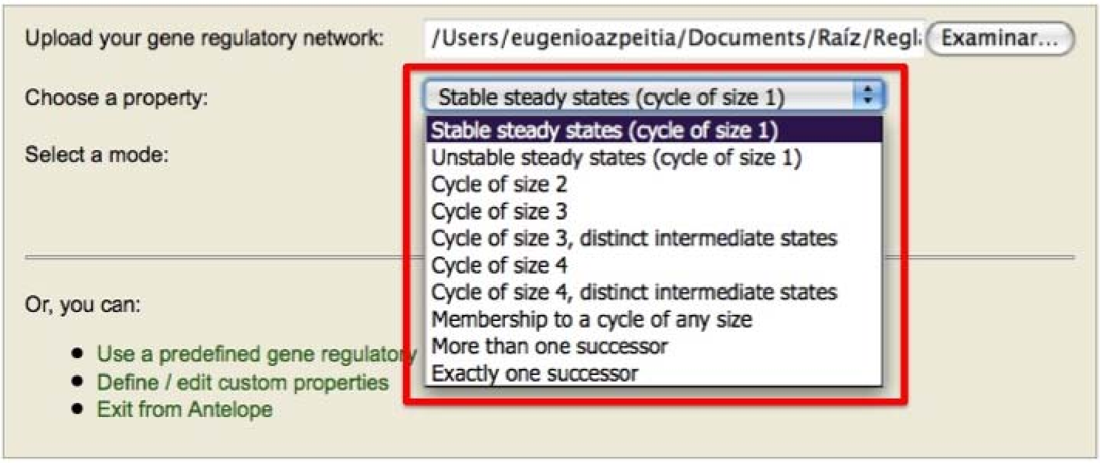

Figure 4. Selecting a query. The drop-down list of properties is framed in red.

2.2 Using preloaded formulas from the results screen

After getting the results of the first query it is possible to make a new one. Use the drop-down list of the results screen (figure 5) and click on the “submit” button.

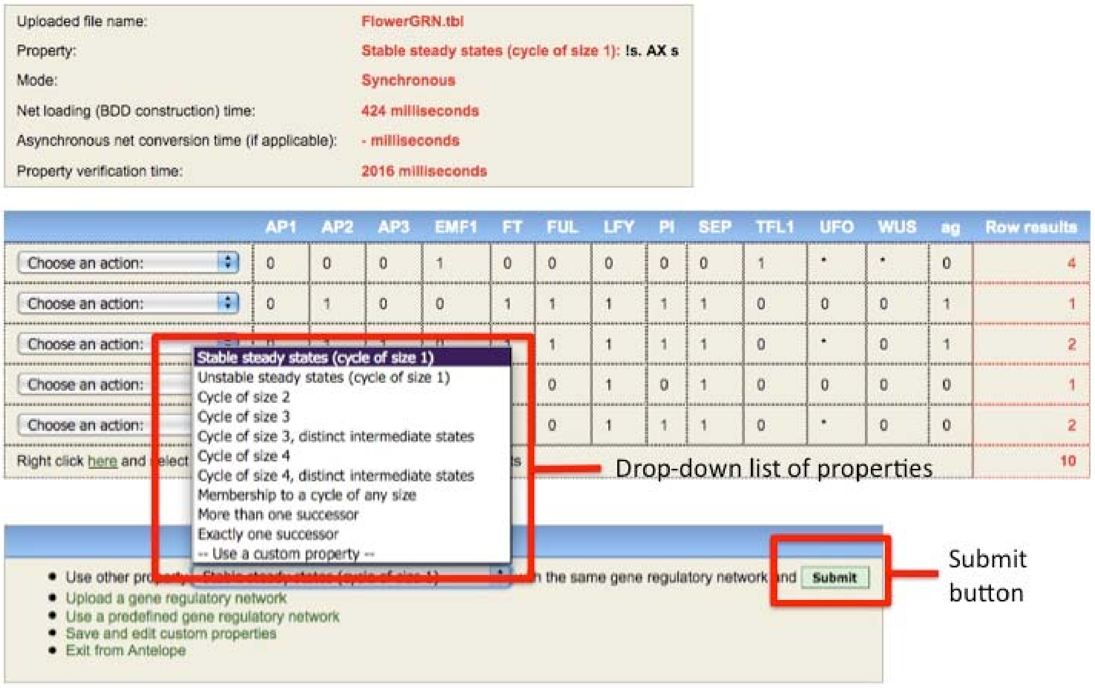

Figure 5. Selecting a query from the results screen

2.3 Using preloaded formulas from the formula properties screen

To use a preloaded property from the formula properties screen click on a formula (the formulas screen is shown in figure 6).

3. Saving your results

It is possible to save the results of any query by clicking "here" in the legend of the results screen "Right click here and select 'Save link as' to get a plain text file with this results" (see figure 7).

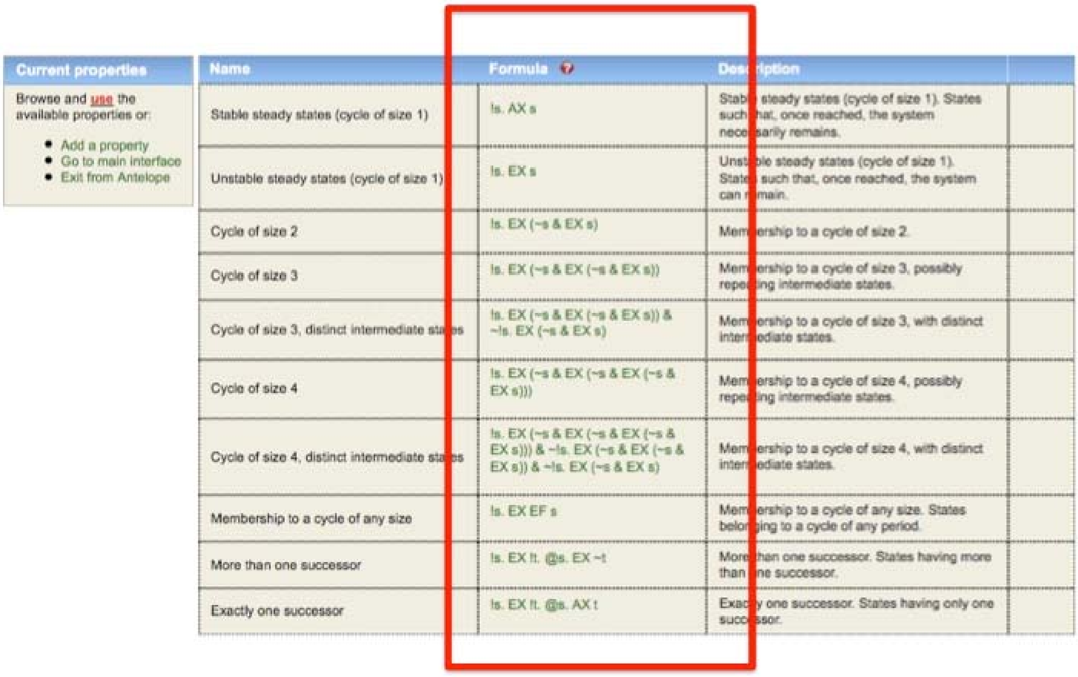

Figure 6. Recalculating a property from the formula properties screen. Formulas are framed in red.

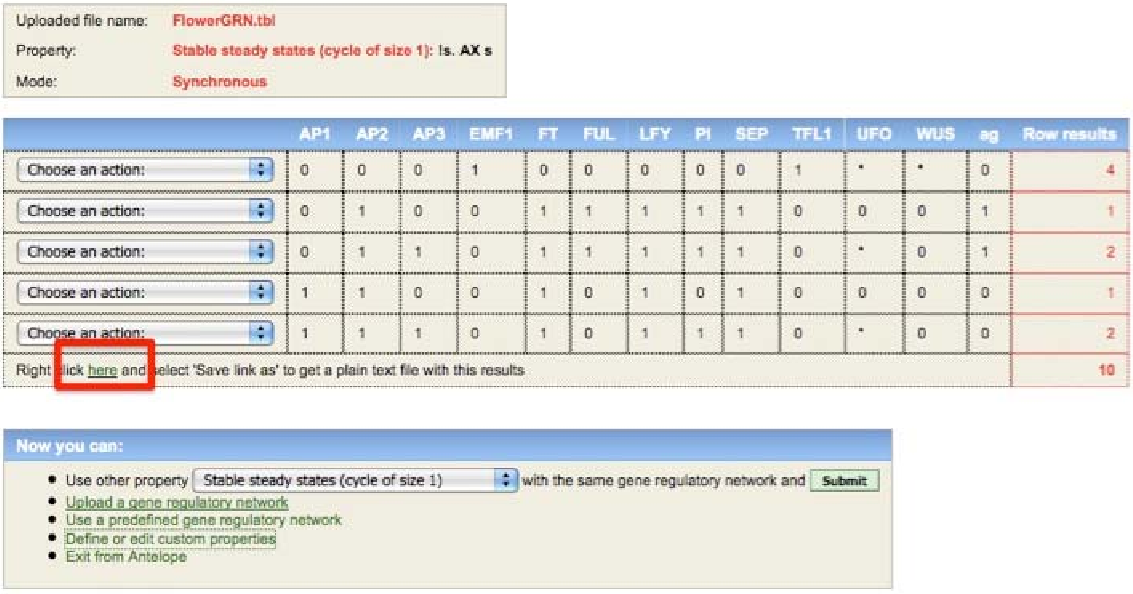

Figure 7. Saving your results. The “here” option is framed in red.

4. Synchrony and Asynchrony

Antelope can handle three types of network state change, each allowing to explore different dynamic properties of network under study. These state changes are the Synchronous, Asynchronous and Synchronous-Asynchronous. You can choose from these three options before you submit a query by clicking the desired option in the drop-down list that appears in the figure 8. The way each state change operates is shown below in figure 9.

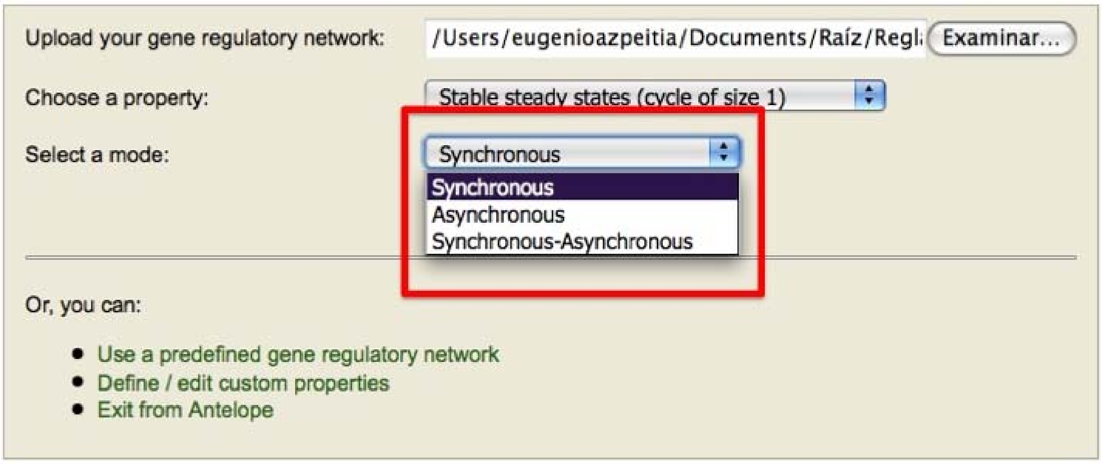

Figure 8. Synchrony and asynchrony

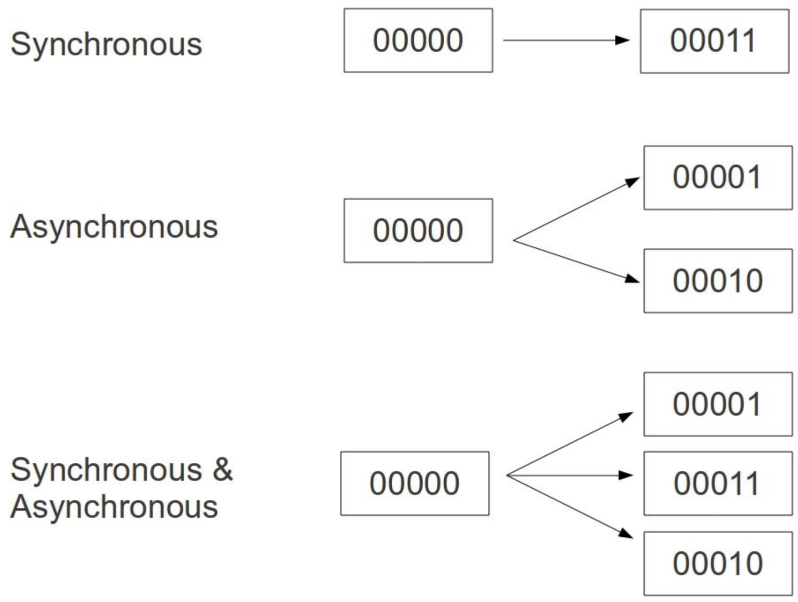

Figure 9. Three types of state change for the last two genes.

5. Define or edit custom properties

We believe that the preloaded formulas represent important properties for inspecting a model. The information of these properties is presented next to them in the formula properties screen (figure 10). The “Name” field is an abbreviated form to designate the formula we are using. The “Formula” field presents the formula used in HCTL syntax. The “Description” field contains additional information about a property.

Further information regarding the HCTL syntax can be obtained through the “Edit and create properties” screen. To access this screen click on the “Add a property” button (figure 10).

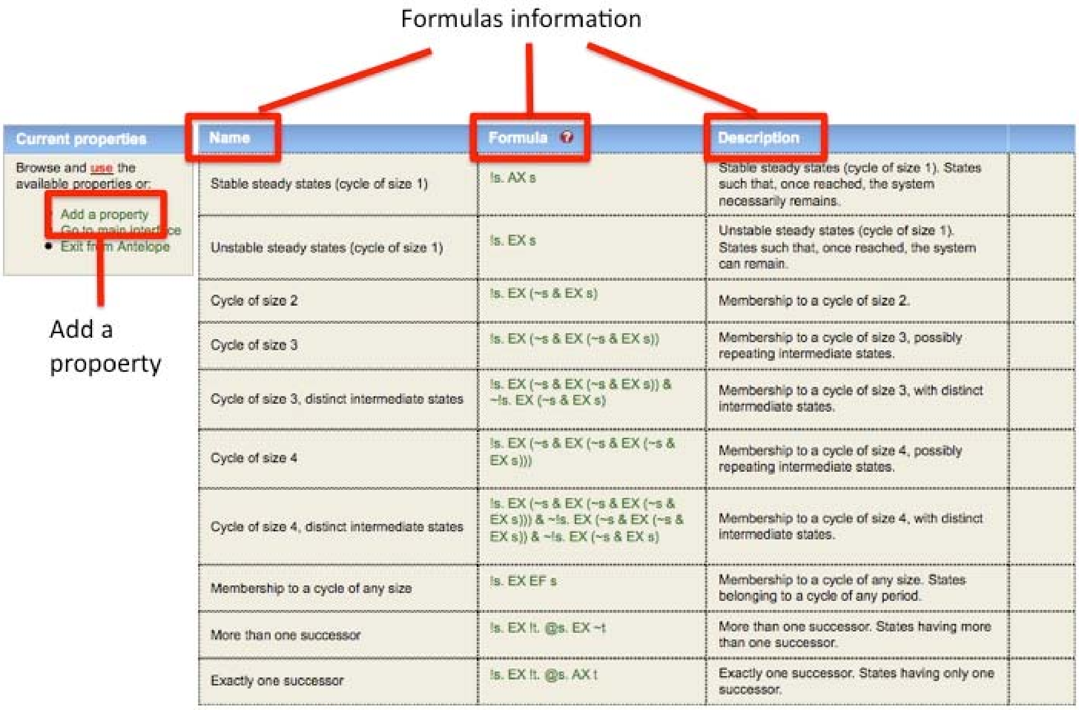

Figure 10. The information fields of the formulas and the add property option.

5.1 Add a new property

Antelope allows adding new properties expressed in HCTL. To add a new property click on the "add a property" option in the formula properties screen (figure 10). Then the "define and edit custom properties" screen appears (figure 11). In this screen you may type the three information fields: "Name", "Formula" and "Description" with the information explained above (figure 11). The "Formula" field is associated with a parser that will let you known if a mistake was made as the formula is typed (figure 12). After the information field is filled up, click on "add property" (figure 11). Antelope saves every new formula, so once you have added it, you will find it every time you access Antelope.

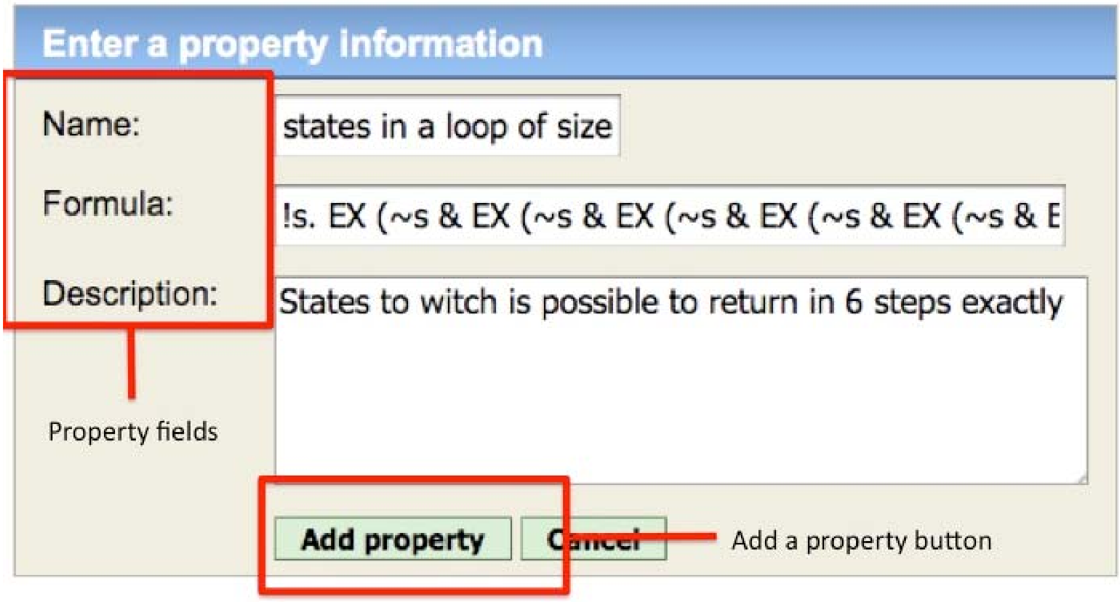

Figure 11. Adding a new property.

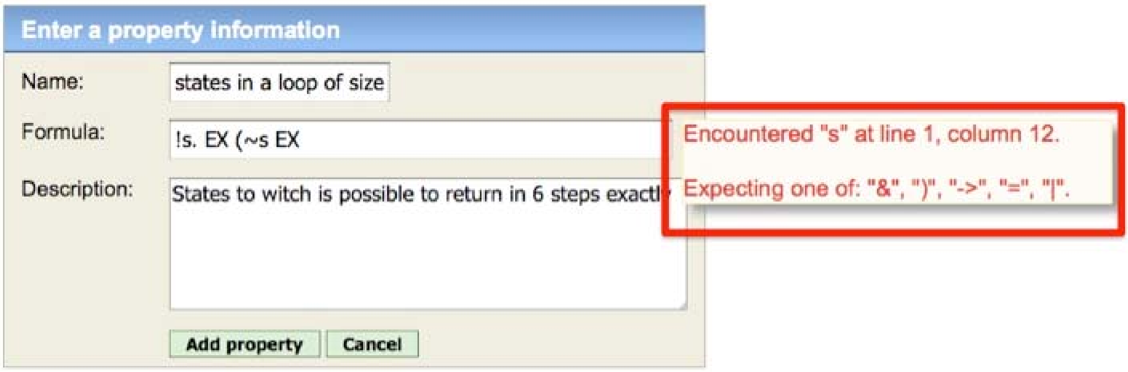

Figure 12. Mistake message displayed when adding a new property have syntax errors. The message is framed in red.

5.2 Remove a property

It is possible to remove the custom formulas with the "Remove" option (figure 13).

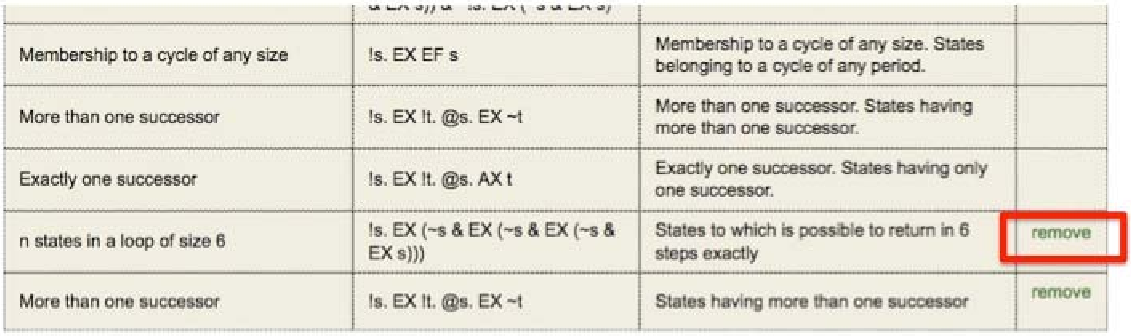

Membership to a cycle of any size	!s. EX EF s	Membership to a cycle of any size. States belonging to a cycle of any period.	
More than one successor	!s. EX !t. @s. EX ~t	More than one successor. States having more than one successor.	
Exactly one successor	!s. EX !t. @s. AX t	Exactly one successor. States having only one successor.	
n states in a loop of size 6	!s. EX (~s & EX (~s & EX (~s & EX s)))	States to which is possible to return in 6 steps exactly	remove
More than one successor	!s. EX !t. @s. EX ~t	States having more than one successor	remove

Figure 13. Removing a property. The “remove” button is framed in red.

6. Manipulating the results

6.1 Wildcards, basin of attraction, successors and predecessors

When possible, Antelope abbreviates the results with wildcards (* indicates that the state of the corresponding gene can be either 0 or 1). This abbreviation can be overridden with the option “remove wildcards” of the “Choose an action” drop-down list (figure 14). The same list has the option to calculate basins of attraction, successors and predecessors.

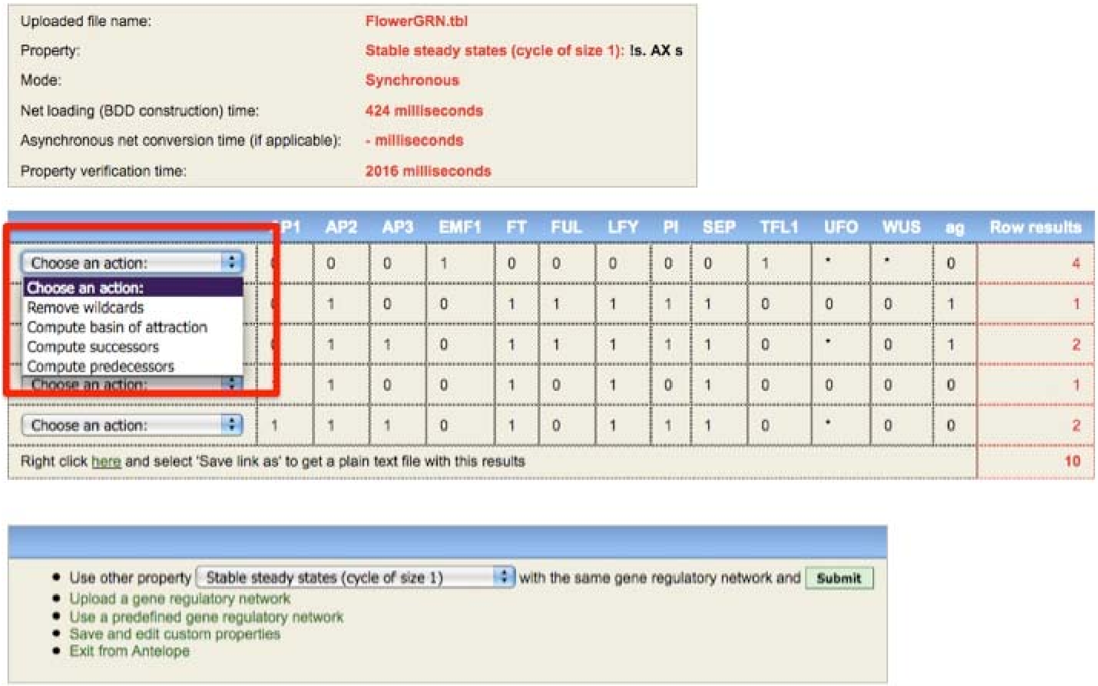

Figure 14. Working with or without wildcards. The drop-down list “Choose an action” button is framed in red.

MODEL FILE SYNTAX

A model can be specified in either of two ways, by means of **tables** or by means of **logic statements**. For distinguishing between these model representations, Antelope requires two distinct file extensions:
(a) `.tbl` for table model representations, and
(b) `.eqn` for logic statements model representations.

In the table format, each table defines the value of a different gene in the next time step as a function of the current values of other genes. Each table has two parts: a head with the names of the genes, followed by a body, with the values of the genes.

The head of a table consists of: (a) the name of the gene, say Z, whose behavior is specified by that table, (b) one or more underscores, and (c) the names of the genes, say X1,…,Xn. In such a table, the values of X1,…,Xn at time t determine the value of Z at time t+1.

The body of a table consists of rows. Each row, unlike the head of the table, first lists the current values of X1,…,Xn (at time t), and then the value of Z (at time t+1). The current values of X1,…,Xn must appear in the same order as in the head of the table.

In each row there is a bar "|" in between the current values of X1,…,Xn and the value of Z. Values can be 0, 1 or *. A fragment of a model would be:

```
PLT____________________ARF
   0|0
   1|1
AUXINAS____________________AUXINAS
   0|*
   1|1
IAA____________________AUXINAS
   0|1
   1|0
ARF____________________IAA
   0|1
   1|0
SHR____________________SHR
   0|0
   1|1
SCR____________________SHR SCR JKD MGP
   0000|0
   0001|0
   0010|0
   0011|0
   0100|0
   0101|0
   0110|0
   0111|0
   1000|0
```

1001|0
1010|1
1011|1
1100|1
1101|0
1110|1
1111|1

In the logic statements format, the file consists of a list of logic equations in either of the following two forms:

Z := f; (1)

Z := f, g; (2)

where Z is the name of the gene we are specifying, and f and g are Boolean formulas describing the conditions that activate Z.

In case there are no indeterminations form (1) is used. In case of indeterminations, however, form (2) is used specifying two functions: f and g. The function f describes Z when the value of all indeterminations for Z is zero. The function g, by contrast, describes Z when the value of all indeterminations is one. For example:

x	y	x'
0	0	*
0	1	0
1	0	1
1	1	*

We first rewrite this table as two tables, one in which * corresponds to 0, and another in which * corresponds to 1:

x	y	x'
0	0	0
0	1	0
1	0	1
1	1	0

x	y	x'
0	0	1
0	1	0
1	0	1

1 1 | 1

In this case

f = x & ~y

and

g = x | (~x & ~y)

finally, we get:

x := x & ~y, x | (~x & ~y)

The formulas f and g have the following syntax:

Boolean operation	Antelope's syntax
T	true
⊥	false
¬ φ	~ φ
φ ∧ ψ	φ & ψ
φ ∨ ψ	φ | ψ
φ ⇒ ψ	φ –> ψ
φ ⇔ ψ	φ = ψ

As an example, consider the following logic statements equivalent to the table-based model above:

```
/* logic statement-based model example */

PLT := ARF;

AUXINAS := true, AUXINAS;

IAA := ~AUXINAS;

ARF := ~IAA;

SHR := SHR;

SCR := SHR & ~SCR &  JKD & ~MGP |
```

```
SHR & ~SCR &  JKD &  MGP |
SHR &  SCR & ~JKD & ~MGP |
SHR &  SCR &  JKD & ~MGP |
SHR &  SCR &  JKD &  MGP;
```

GENE NAMES

Gene names may contain arbitrary alphanumeric characters. However, for avoiding name collisions, the names of genes should be different from the names of the temporal operators.

If the user has a gene name which coincides with an operator name, it is possible to circumvent this collision by renaming the gene with lower-case letters, for example. For instance, if a gene is called "AG", which is also the name of a temporal operator, such a gene could be renamed as "ag".

FORMULAS

Formulas in Antelope have the following syntax:

Hybrid CTL	Antelope's syntax
T	true
$\perp$	false
$\neg\varphi$	$\sim\varphi$
$\varphi \wedge \psi$	$\varphi \,\&\, \psi$
$\varphi \vee \psi$	$\varphi \mid \psi$
$\varphi \Rightarrow \psi$	$\varphi \text{->} \psi$
$\varphi \Leftrightarrow \psi$	$\varphi = \psi$
AX φ	AX φ
EX φ	EX φ
AY φ	AY φ
EY φ	EY φ
A[φUψ]	A(φUψ)
E[φUψ]	E(φUψ)
EF φ	EF φ
EG φ	EG φ
AF φ	AF φ
AG φ	AG φ
@σ. φ	@s. φ
$\downarrow\sigma$. φ	! s. φ
$\exists\sigma$. φ	] s. φ

For example:
Hybrid CTL:

$$AG(\ p \vee q \vee r \Rightarrow EF\ EG\ r)$$

Antelope's syntax:

$$AG(\ p \mid q \mid r \text{ -> } EF\ EG\ r\ )$$

Also, the following table shows the precedence of operators (the higher the precedence, the tighter the operator is attracted to its arguments):
The models can have one-line comments beginning with "//", and multi-line comments enclosed between "/*" and "*/".

Precedence	Operator
5	~, @, !,], AX, EX, AF, EF, AG, EG, A(U), E(U), AY, EY
4	&
3	|
2	->
1	=

The algorithm used by *Antelope* for checking a GRN with respect to CTL formulas is efficient (taking polynomial time in the size of the Kripke structure). The additional expressiveness of hybrid operators, such as "!" comes at a price, however. Given a CTL formula φ, the computation of the set of states at which a formula of the form "! s . φ" holds involves calling the CTL model-checking algorithm with φ *once for each state*. The decrease in efficiency is even more if the "!" operator appears nested. Although *Antelope* treats certain patterns in special ways, requiring less time than a direct approach, formulas using hybrid operators may take an excessive time to check.

Finally, in Antelope there are two different ways to specify a networks state in a formula: 1) as a minterm, or 2) as a nominal. Nominals are numeric representations of network states. Antelope can handle three different nominal codifications, which are binary, decimal, and hexadecimal. The binary codification uses the prefix "0b" or "0B", and the hexadecimal codification the prefix "0x" or "0X". Note that for binary, decimal, and hexadecimal codifications, Antelope arranges the genes following the ASCII lexicographic character order.

For example, assume we have a network with two nodes, namely "geneA" and "geneB", and that we want to specify the network state where "geneA" is expressed and "geneB" is not expressed. As a minterm, this network state is represented with the formula "geneA & ~geneB". The same state is represented as "0b10", "2", and "0x2" in a binary, decimal and hexadecimal codification respectively.

REFERENCES

[1] Espinosa-Soto C, Padilla-Longoria P, Alvarez-Buylla ER. A gene regulatory network model for cell-fate determination during Arabidopsis thaliana flower development that is robust and recovers experimental gene expression profiles. Plant Cell. 2004, 16:2923-39.

[2] Azpeitia et al. Single-cell and coupled GRN models of cell patterning in the Arabidopsis thaliana root stem cell niche. BMC Systems Biology 2010, 4:134.
